# Proteogenomic analysis reveals cytoplasmic sequestration of RUNX1 by the acute myeloid leukemia–initiating CBFB::MYH11 oncofusion protein

**DOI:** 10.1172/JCI176311

**Published:** 2023-12-07

**Authors:** Ryan B. Day, Julia A. Hickman, Ziheng Xu, Casey D.S. Katerndahl, Francesca Ferraro, Sai Mukund Ramakrishnan, Petra Erdmann-Gilmore, Robert W. Sprung, Yiling Mi, R. Reid Townsend, Christopher A. Miller, Timothy J. Ley

**Affiliations:** 1 Section of Stem Cell Biology, Division of Oncology, Department of Internal Medicine, and; 2 Division of Endocrinology, Metabolism and Lipid Research, Department of Internal Medicine, Washington University School of Medicine, St. Louis, Missouri, USA.

**Keywords:** Oncology, Epigenetics, Leukemias, Proteomics

## Abstract

Several canonical translocations produce oncofusion genes that can initiate acute myeloid leukemia (AML). Although each translocation is associated with unique features, the mechanisms responsible remain unclear. While proteins interacting with each oncofusion are known to be relevant for how they act, these interactions have not yet been systematically defined. To address this issue in an unbiased fashion, we fused a promiscuous biotin ligase (TurboID) in-frame with 3 favorable-risk AML oncofusion cDNAs (*PML::RARA*, *RUNX1::RUNX1T1*, and *CBFB::MYH11*) and identified their interacting proteins in primary murine hematopoietic cells. The PML::RARA- and RUNX1::RUNX1T1-TurboID fusion proteins labeled common and unique nuclear repressor complexes, implying their nuclear localization. However, CBFB::MYH11-TurboID–interacting proteins were largely cytoplasmic, probably because of an interaction of the MYH11 domain with several cytoplasmic myosin-related proteins. Using a variety of methods, we showed that the CBFB domain of CBFB::MYH11 sequesters RUNX1 in cytoplasmic aggregates; these findings were confirmed in primary human AML cells. Paradoxically, *CBFB::MYH11* expression was associated with increased *RUNX1/2* expression, suggesting the presence of a sensor for reduced functional RUNX1 protein, and a feedback loop that may attempt to compensate by increasing *RUNX1/2* transcription. These findings may have broad implications for AML pathogenesis.

## Abstract 2

<p>The CBFB::MYH11 fusion protein that initiates Acute Myeloid Leukemia sequesters RUNX1 in the cytoplasm, reducing its activity&nbsp;and initiating a feedback loop&nbsp;that increases RUNX1 expression.</p>

## Graphical abstract

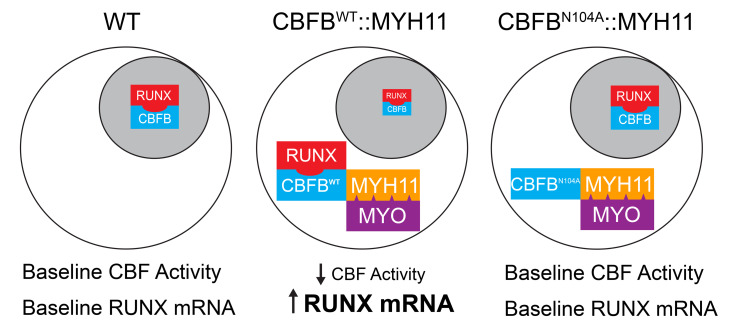

## Introduction

Oncofusion genes exist in a variety of solid tumors and hematopoietic malignancies, and are often predictive of outcomes. Elucidating the mechanism of action of an oncofusion protein presents unique challenges, since they are not present in normal cells, and may have loss-of-function, gain-of-function, or neomorphic properties relative to the normal roles of their constituent components. Oncofusion genes are common initiating events for acute myeloid leukemia (AML), found in approximately 20%–40% of AML cases (1–3). Three oncofusions are included in the favorable risk subgroup of AML: *PML::RARA* AML is characterized by a fusion between PML, a nuclear protein with a variety of functions, and retinoic acid receptor α (RARA), a nuclear receptor and transcription factor (4–7). Fusion of the gene encoding the transcription factor RUNX1 with the gene encoding the corepressor RUNX1T1 defines *RUNX1::RUNX1T1* AML (8–11). The fusion of the gene encoding the transcription factor CBFB with the gene encoding smooth muscle myosin heavy chain 11 (MYH11) initiates *CBFB::MYH11* AML (12–18). RUNX1 (or its paralog RUNX2 or RUNX3) and CBFB heterodimerize in a 1:1 ratio to form core binding factor (CBF), a transcription factor that is essential for hematopoiesis (19–32); for this reason, *RUNX1::RUNX1T1* and *CBFB::MYH11* AMLs are commonly considered together as core binding factor–AMLs (CBF-AMLs). *RUNX1::RUNX1T1* is thought to initiate AML through its function as a transcriptional repressor, recruiting repression machinery to genomic CBF binding sites, downregulating CBF target genes (33–37). The mechanisms by which *CBFB::MYH11* initiates AML have been harder to define (38). One widely accepted model proposes that CBFB::MYH11 functions similarly to RUNX1::RUNX1T1, recruiting transcriptional repressor machinery to genomic CBF binding sites, via the MYH11 domain (33, 39–42). However, several lines of evidence suggest that CBFB::MYH11 and RUNX1::RUNX1T1 may act by different mechanisms. AMLs with these fusions have distinct DNA methylation patterns (43–45), distinct gene expression profiles (43, 46), distinct proteomic signatures (47), different common cooperating mutations (43, 48–52), and different clinical phenotypes (53–59). Other proposed models of *CBFB::MYH11* AML initiation take some of these differences into account. One suggests that CBFB::MYH11 may sequester RUNX1 from WT CBFB, reducing the CBF activity (60–62). Another suggests that the inactivation of one *CBFB* allele by the *CBFB::MYH11* fusion causes CBFB haploinsufficiency, and that reduced CBFB function is relevant to pathogenesis (63).

Proximity biotin ligase labeling is a recently developed technology that permits unbiased detection of protein interactions in living cells (64, 65). TurboID is a highly active, promiscuous biotin ligase that biotinylates proteins within 10–15 angstroms (66); any protein fused in-frame with TurboID can therefore act as a “bait” for its normal interacting proteins, allowing for their biotinylation, purification, and identification by Western blotting or mass spectrometry. In contrast to antibody-mediated pull-downs, TurboID relies on high-affinity biotin-streptavidin interactions, allowing for the use of very stringent wash steps that minimize nonspecific background. Further, this method does not rely on antibody specificity to identify the bait protein, is not highly sensitive to buffer composition, and does not require an interaction to be occurring exactly at the time of cell lysis, since biotin labeling occurs over a period of hours. Importantly, since biotin labeling occurs in living cells, protein interactions occur in vivo in the defined cellular compartments (e.g., nuclear or cytoplasmic) in which proteins execute their normal biological functions. Proximity labeling is especially attractive for oncofusion proteins, since antibodies directed at one component of the oncofusion will also often interact with its WT counterpart. Although it is sometimes possible to develop antibodies against a unique fusion sequence, breakpoints are often heterogeneous, meaning that different antibodies may be needed to evaluate every fusion type.

In this study, we examined the protein interactomes of the 3 favorable-risk AML oncofusion proteins, PML::RARA, RUNX1::RUNX1T1, and CBFB::MYH11, using primary murine hematopoietic cells transduced with a retroviral TurboID vector system developed in our laboratory. While PML::RARA and RUNX1::RUNX1T1 primarily interacted with proteins associated with well-described nuclear complexes, CBFB::MYH11 had a distinct interactome characterized by interaction with myosin-related proteins, which are normally located in the cytoplasm. Based on these data, we developed orthogonal approaches that revealed that CBFB::MYH11 is primarily cytoplasmic in primary murine hematopoietic cells and primary human *CBFB::MYH11* AML cells, and that the fusion protein sequesters RUNX1 in cytoplasmic aggregates, probably reducing the activity of CBF in the nucleus. Further study of primary human AML samples revealed that dysregulation of *CBFB* and *RUNX1* expression was detected in all tested human AMLs, suggesting that altered CBF activity may be a more general feature of AML biology than previously suspected. We provide an approach for utilizing proximity labeling in primary hematopoietic cells, and highlight the value of protein interactome studies for the study of protein-protein interactions in living, primary cells.

## Results

### TurboID proximity labeling in primary murine hematopoietic stem and progenitor cells.

**Figure 1:**
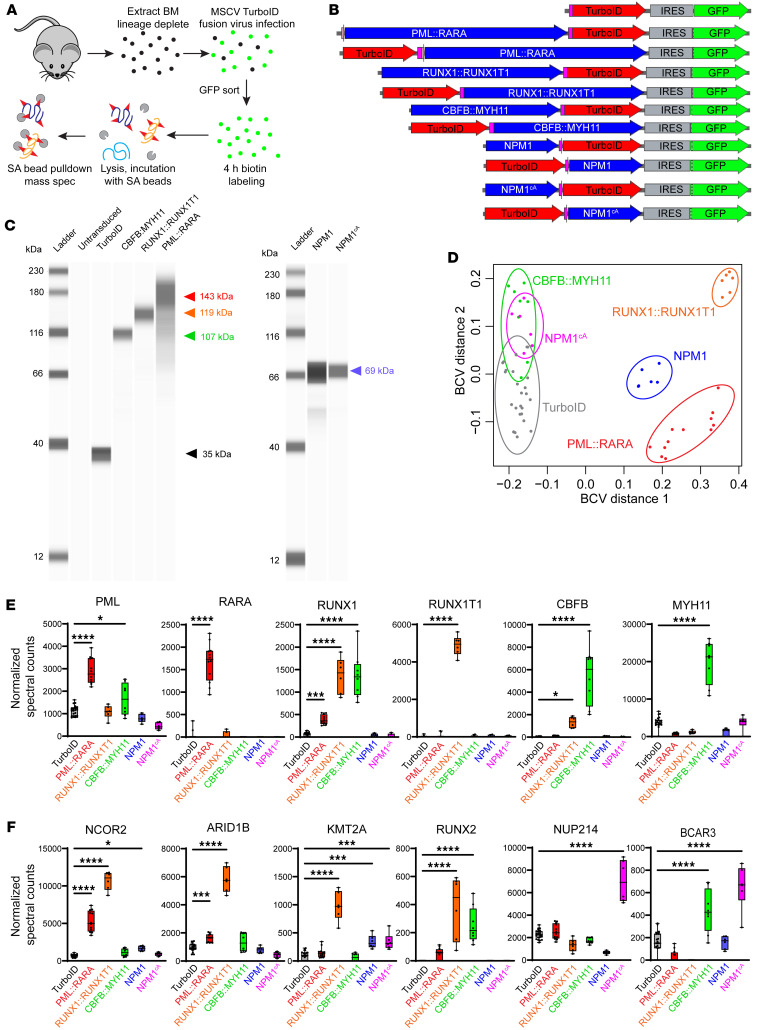
Overview of TurboID proximity labeling experiments in primary murine hematopoietic cells. (**A**) Experimental schema for TurboID experiments. WT murine bone marrow was harvested, lineage-depleted, and transduced with MSCV retrovirus encoding TurboID alone or fused to a gene of interest. GFP^+^ cells were sorted to enrich for transduced cells, which were incubated in biotin-containing medium to facilitate TurboID-mediated labeling of proximate proteins. Cells were lysed, and lysates were incubated with streptavidin (SA) beads. Proteins were eluted from washed beads and identified by mass spectrometry. (**B**) Schematic of vectors used in TurboID experiments. (**C**) Gel images from ProteinSimple Jess blots showing size of TurboID fusion proteins detected with an anti-TurboID antibody. Arrowheads indicate expected sizes (black, TurboID alone; red, PML::RARA; orange, RUNX1::RUNX1T1; green, CBFB::MYH11; blue, NPM1 or NPM1^cA^). (**D**) Multidimensional scaling plot of TurboID data with the indicated TurboID fusion proteins. Note overlap between CBFB::MYH11- and NPM1^cA^-TurboID interactomes. TurboID, *n* = 23; PML::RARA, *n* = 12; RUNX1::RUNX1T1, *n* = 6; CBFB::MYH11, *n* = 8; NPM1, *n* = 6; NPM1^cA^, *n* = 6. BCV, biological coefficient of variation. (**E**) Normalized spectral counts for the indicated oncofusion protein components. TurboID fusion proteins are self-biotinylating, and oncofusion proteins are detected in transduced cells. One-way ANOVA relative to TurboID alone, *P < 0.05, ***P < 0.001, ****P < 0.0001, nonsignificant comparisons not labeled. Each point represents 1 sample, bar indicates mean, box indicates 95% confidence interval, whiskers indicate value range. (**F**) Selected TurboID-biotinylated target proteins with significant interactions with oncofusion “bait” proteins. The proteins displayed were chosen based on previously reported interactions, to demonstrate platform accuracy. BCAR3 is a documented cytoplasmic protein that interacts with both NPM1^cA^ and CBFB::MYH11, suggesting that they are also predominantly located in the cytoplasm. One-way ANOVA relative to TurboID alone, *P < 0.05, ***P < 0.001, ****P < 0.0001, nonsignificant comparisons not labeled.

To better understand mechanisms of transformation in fusion oncogene–initiated AML, we used the TurboID proximity ligase system to identify interacting proteins of 3 major favorable-risk oncofusions. TurboID is a highly active, promiscuous biotin ligase that can biotin-label proteins in intact, living cells, within 15 angstroms of a “bait” protein fused to TurboID. Biotinylated proteins can be enriched from whole-cell lysates using streptavidin beads and identified via mass spectrometry (Figure 1A). We fused *TurboID* cDNA to the 5′ or 3′ end of oncofusion cDNAs encoding *PML::RARA*, *RUNX1::RUNX1T1*, or *CBFB::MYH11* in the retroviral vector MSCV-IRES-GFP (Figure 1B), so that we could express these proteins in primary hematopoietic progenitor cells. *TurboID* alone serves as a control for the labeling of proteins that are not supervised by the fusion oncoprotein. Nucleophosmin 1 (*NPM1*) is a frequently mutated AML gene, and the most common mutation (*NPM1^cA^*) results in translocation of the protein from its usual nucleolar location to the cytoplasm. We previously reported data comparing the NPM1 and NPM1^cA^ protein interactomes in mouse hematopoietic cells using the TurboID system (47), and we have included these data sets here as additional reference groups. We extracted bone marrow from 8- to 16-week-old WT C57BL/6J mice, enriched for hematopoietic stem and progenitor cells (HSPCs) with lineage depletion, and transduced these cells with MSCV-IRES-GFP–based retroviruses encoding either TurboID alone or TurboID fused to an AML oncofusion protein. Two days later, cells were sorted for GFP to enrich for the transduced population, and cultured for 2 additional days. These intact, GFP^+^ cells were incubated with biotin for 4 hours and lysed. Protein lysates were incubated with streptavidin beads overnight to allow binding of biotinylated proteins to streptavidin beads, which were then stringently washed. Bound proteins were eluted from streptavidin beads with trypsin digestion and identified by mass spectrometry.

Western blotting using a TurboID-specific antibody showed fusion proteins of the expected sizes in the transduced cells (Figure 1C); N- and C-terminal fusions were similarly sized (data not shown). Multidimensional scaling plots based on the biological coefficient of variation were used to assess reproducibility across experiments (Figure 1D). PML::RARA-, RUNX1::RUNX1T1-, and NPM1-TurboID fusion samples each formed unique clusters. Unexpectedly, the CBFB::MYH11-TurboID cluster was more similar to that of NPM1^cA^-TurboID than to that of RUNX1::RUNX1T1-TurboID, suggesting a very different set of interacting proteins. N- and C-terminal fusions behaved similarly (Supplemental Figure 1; supplemental material available online with this article; https://doi.org/10.1172/JCI176311DS1) and were therefore combined for subsequent analyses.

TurboID is self-biotinylating, so any protein fused to TurboID should be biotin-labeled and pulled down with streptavidin beads. As expected, we detected biotin-labeled PML and RARA in cells expressing PML::RARA-TurboID, RUNX1 and RUNX1T1 in cells expressing RUNX1::RUNX1T1-TurboID, and CBFB and MYH11 in cells expressing CBFB::MYH11-TurboID (Figure 1E). Consistent with previously reported studies, we detected interactions of RUNX1::RUNX1T1 and PML::RARA with multiple transcriptional repressors (7, 36); the labeling of 3 representative proteins (NCOR2, ARID1B, and KMT2A) is shown in Figure 1F. As expected based on CBFB heterodimerization with RUNX-family proteins, CBFB::MYH11 interacted with both RUNX1 (Figure 1E) and RUNX2 (Figure 1F). As previously reported (47), NPM1^cA^ interacted with multiple nuclear pore proteins, and one representative example (NUP214) is shown in Figure 1F. Interestingly, both NPM1^cA^ and CBFB::MYH11 interacted with BCAR3, a protein previously reported to be restricted to the cytoplasm/cell membrane (67–69), suggesting that CBFB::MYH11-TurboID is primarily cytoplasmic in this system. All identified interacting proteins for each TurboID fusion protein can be queried at https://leylab.org/Favorable_Risk_AML_TurboID.html

### AML oncofusions have distinct protein interactomes.

**Figure 2:**
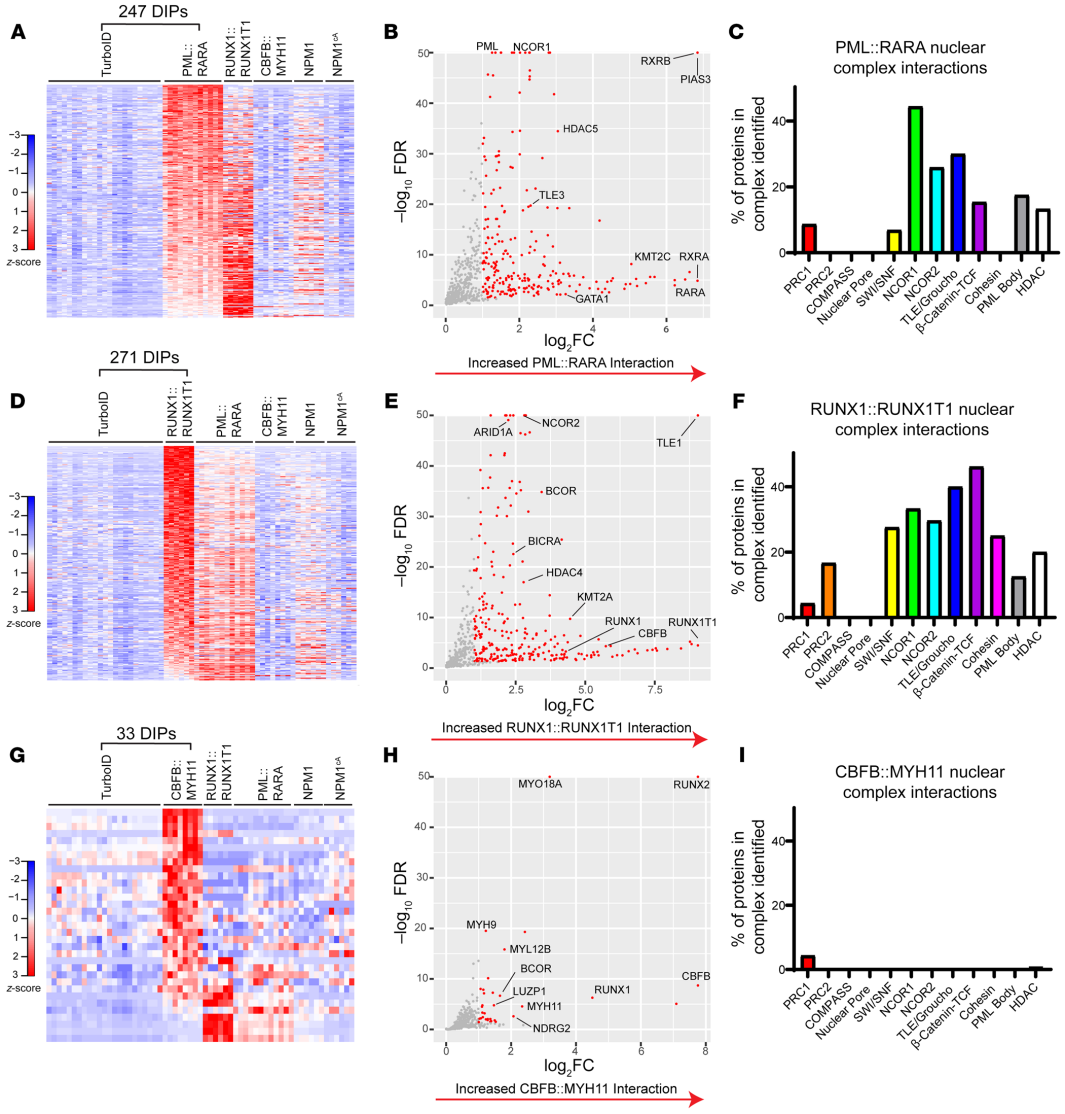
PML::RARA, RUNX1::RUNX1T1, and CBFB::MYH11 have distinct protein interactomes in mouse hematopoietic cells. (**A**) Heatmap showing differentially interacting proteins (DIPs) (edgeR, FDR < 0.05, >2-fold change) with increased detection in PML::RARA-TurboID fusion samples (*n* = 12) relative to TurboID-alone samples (*n* = 23); the same proteins detected with the other TurboID fusion samples are plotted “passively.” Data are shown as *z*-scored normalized spectral counts. (**B**) Volcano plot of proteins identified in **A**, with selected proteins labeled. (**C**) The percentage of proteins in selected nuclear complexes with detectable interactions with PML::RARA-TurboID fusion, relative to TurboID alone. (**D**) Heatmap showing DIPs with increased detection in RUNX1::RUNX1T1-TurboID fusion samples (*n* = 6) relative to TurboID-alone samples (*n* = 23). (**E**) Volcano plot of proteins identified in **D**, with selected proteins labeled. (**F**) The percentage of proteins in selected nuclear complexes with detectable interactions with RUNX1::RUNX1T1-TurboID fusion, relative to TurboID alone. (**G**) Heatmap showing DIPs with increased detection in CBFB::MYH11-TurboID fusion samples (*n* = 8), relative to TurboID-alone samples (*n* = 23). (**H**) Volcano plot of proteins identified in **G**, with selected proteins labeled. (**I**) The percentage of proteins in selected nuclear complexes with increased interaction with CBFB::MYH11-TurboID fusions, relative to TurboID alone.

We next identified differentially interacting proteins (DIPs) for each oncofusion protein compared with TurboID alone (significance defined as a more than 2-fold increased interaction with the oncofusion-TurboID protein relative to TurboID alone, FDR < 0.05). PML::RARA-interacting proteins were very similar across biological replicates, with 247 DIPs identified relative to TurboID alone (Figure 2, A and B, and Supplemental Table 1); a subset of these proteins also interacted with RUNX1::RUNX1T1, but not CBFB::MYH11. PML::RARA interacted with a number of proteins in well-defined nuclear complexes (Supplemental Table 2), including “PML bodies” and nuclear repressor complexes (NCOR1, NCOR2, TLE/Groucho, HDAC; Figure 2C). Similarly, RUNX1::RUNX1T1 had a highly canonical set of 271 DIPs (Figure 2, D and E, and Supplemental Table 3), and a subset of these proteins also interacted with PML::RARA, but not CBFB::MYH11. RUNX1::RUNX1T1 specifically interacted with a variety of chromatin modifier and transcriptional repressor proteins (Figure 2F), consistent with its proposed mechanism of action as a nuclear corepressor. Interestingly, RUNX1::RUNX1T1 also interacted with proteins in the cohesin complex (STAG1, STAG2, and RAD21); although mutations in genes encoding cohesin complex proteins commonly occur in *RUNX1::RUNX1T1* AML (48–50, 52), direct physical interactions between RUNX1::RUNX1T1 and cohesin proteins have not previously been reported to our knowledge. In contrast, CBFB::MYH11 had essentially no detected interactions with nuclear complex proteins (Figure 2I). While CBFB::MYH11 interacted with RUNX1 and RUNX2, it predominantly interacted with proteins predicted to have a primary cytoplasmic localization, including many myosin and other cytoskeletal proteins (e.g., MYO18A, MYH9, MYL12B, and LUZP1) (Figure 2, G and H, and Supplemental Table 4). Similar studies using the human erythroleukemia cell line K562 also identified interactions between CBFB::MYH11 and myosin-related proteins (data not shown). In contrast to previous studies reporting interaction of CBFB::MYH11 with transcriptional repressors (summarized in Supplemental Table 13), we noted only weak interactions with 2 chromatin modifiers, BCOR and JMJD1C.

### CBFB and CBFB::MYH11 protein interactomes are different.

**Figure 3:**
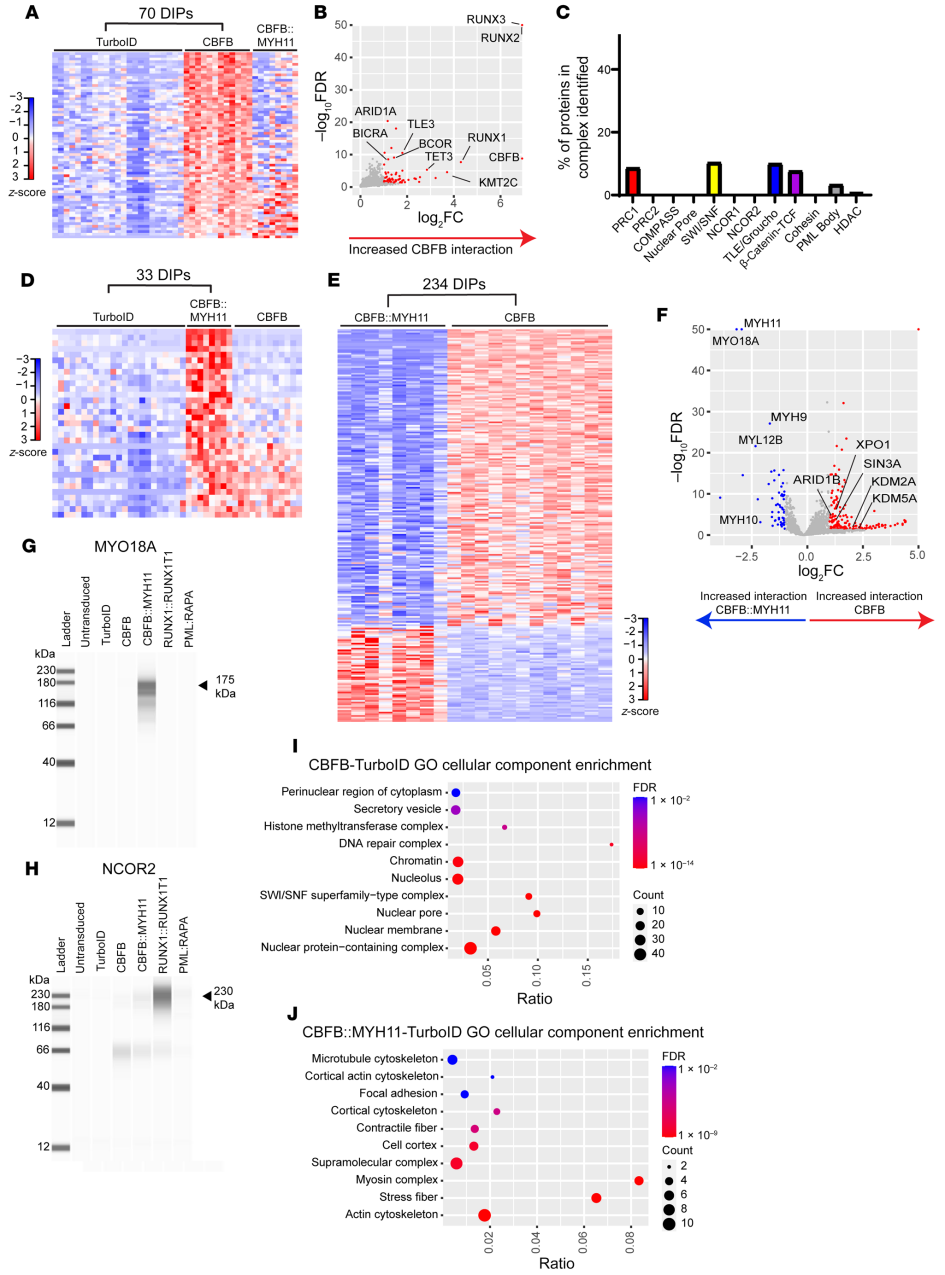
CBFB and CBFB::MYH11 have distinct protein interactomes. (**A**) Heatmap showing DIPs with increased detection in CBFB-TurboID fusion samples (*n* = 12), relative to TurboID alone (*n* = 23); proteins detected with the CBFB::MYH11-TurboID fusion protein are plotted passively. (**B**) Volcano plot of proteins identified in **A**, with selected DIPs labeled. (**C**) Percentage of proteins in selected nuclear complexes with increased interaction with CBFB-TurboID fusion relative to TurboID alone. (**D**) Heatmap showing DIPs with increased detection in CBFB::MYH11-TurboID fusion samples (*n* = 8) relative to TurboID-alone samples, with CBFB-TurboID samples passively plotted. (**E**) Heatmap showing DIPs between CBFB-TurboID and CBFB::MYH11-TurboID fusion proteins. (**F**) Volcano plot of DIPs between CBFB- and CBFB::MYH11-TurboID fusions, with key differential interactors labeled. Myosin-related proteins exclusively interact with CBFB::MYH11, while CBFB interacts predominantly with nuclear proteins. (**G** and **H**) Gel images of ProteinSimple Jess blot on streptavidin beads from HSPCs expressing the indicated TurboID fusions using an antibody against MYO18A (**G**) or NCOR2 (**H**). Note pull-down of MYO18A in CBFB::MYH11-TurboID fusion, and NCOR2 pull-down in RUNX1::RUNX1T1-TurboID fusion. (**I** and **J**) ToppFun pathway enrichment for CBFB-TurboID versus CBFB::MYH11-TurboID DIPs enriched in CBFB-TurboID (**I**) or CBFB::MYH11-TurboID (**J**). Ratio indicates number of genes identified as DIPs divided by number of genes in gene set. Circle size indicates number of proteins identified. FDR, Benjamini-Hochberg FDR.

To define the CBFB::MYH11 interactions mediated by the CBFB moiety of the fusion, we fused *CBFB* cDNA to TurboID and performed similar experiments. *CBFB* isoforms 1 and 2 behaved similarly (data not shown), and were combined for analysis; these full-length cDNAs encoded exons 1–6, while the CBFB portion of CBFB::MYH11 is truncated after exon 5. We identified 70 DIPs between CBFB-TurboID and TurboID alone, including the expected CBFB interactors RUNX1, RUNX2, and RUNX3 (Figure 3, A and B, and Supplemental Table 5). Similarly to PML::RARA and RUNX1::RUNX1T1, CBFB interacted with a number of nuclear complex proteins (Figure 3C), consistent with its function as a component of heterodimeric CBF. Strikingly, most CBFB DIPs (relative to TurboID alone) were not shared with CBFB::MYH11 (Figure 3A), and most CBFB::MYH11 DIPs (relative to TurboID alone) were not shared with CBFB (Figure 3D). We then directly compared CBFB to CBFB::MYH11-interacting proteins and identified 234 DIPs with increased interactions with either CBFB or CBFB::MYH11 (Figure 3E and Supplemental Table 6). CBFB interacted with a variety of nuclear proteins, including nuclear pore proteins and transcriptional regulators, while CBFB::MYH11 predominantly interacted with cytoskeletal proteins, including myosin-related proteins (Figure 3F). We validated the interaction of CBFB::MYH11 with the myosin-related protein MYO18A using Western blotting of proteins enriched with streptavidin beads after biotin labeling (Figure 1A), and confirmed the interaction of MYO18A with CBFB::MYH11-TurboID, but not other TurboID fusions, including CBFB-TurboID (Figure 3G). Using the same method, we confirmed the strong and unique interaction of RUNX1::RUNX1T1 with NCOR2 (Figure 3H). Finally, we performed pathway analysis of DIPs using ToppFun. The most enriched gene ontogeny cellular component gene lists for CBFB-interacting proteins included proteins involved in a variety of nuclear functions (Figure 3I), while CBFB::MYH11-interacting proteins were heavily enriched for cytoplasmic and cytoskeletal functions (Figure 3J), suggesting that CBFB::MYH11 and CBFB have distinct subcellular localization patterns.

### RUNX1 colocalizes with cytoplasmic CBFB::MYH11 in mouse hematopoietic cells.

**Figure 4:**
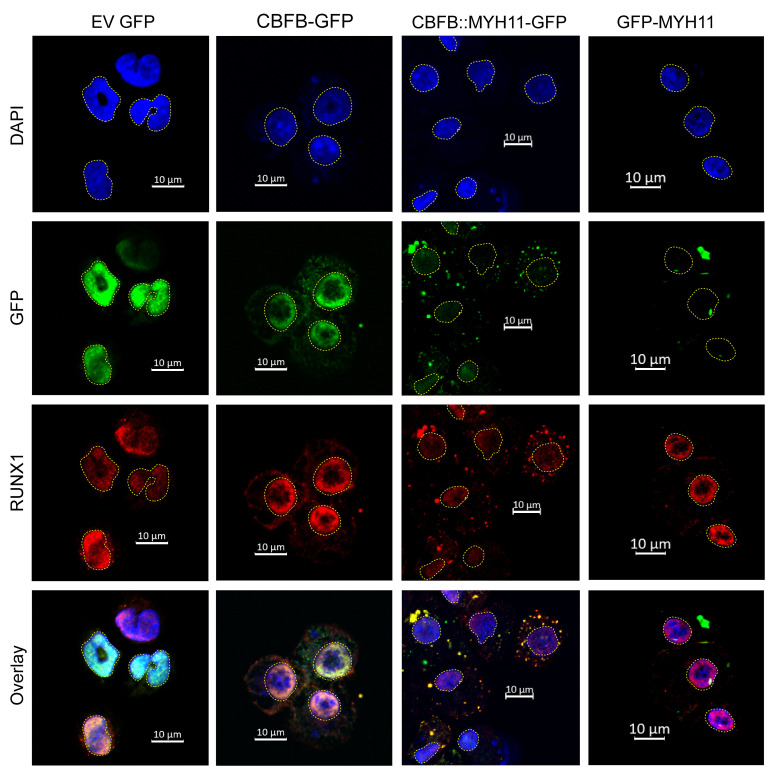
CBFB::MYH11 protein is cytoplasmic in murine hematopoietic cells. MSCV retroviruses were created with GFP fused in-frame with exons 1–5 of *CBFB* (CBFB-GFP), *CBFB::MYH11* (CBFB:GFP-MYH11), or the portion of MYH11 involved in the *CBFB::MYH11* fusion (GFP-MYH11). EV indicates the retrovirus with GFP alone. Lineage-depleted mouse bone marrow cells were transduced with retrovirus and harvested 4–7 days after transduction for immunofluorescence. DAPI staining (identifying the nucleus) is shown in blue, and yellow dotted lines outline the nucleus. GFP (detected directly) is in green, and RUNX1 (detected with antibody staining) is in red. Overlaps between GFP and RUNX1 signals are yellow. In EV-transduced cells, RUNX1 is localized to the nucleus. CBFB-GFP is predominantly localized to the nucleus, where it colocalizes with RUNX1. However, CBFB::MYH11-GFP is predominantly localized in cytoplasmic aggregates; in these cells, RUNX1 is mislocalized from the nucleus to the cytoplasm. GFP-MYH11 forms large aggregates in both the nucleus and the cytoplasm, and does not colocalize with RUNX1. Images shown are representative of 2–4 independent experiments. Scale bars: 10 μm.

The subcellular localization of CBFB::MYH11 in leukemic cells has long been controversial. Most prior studies were performed in nonhematopoietic cells and/or nonprimary tissues, with contradictory results (summarized in Supplemental Table 14). To address this issue, we fused GFP to exons 1–5 of *CBFB* (the exons present in the *CBFB::MYH11* translocation), *CBFB::MYH11*, or exons 33–41 of *MYH11* (the exons present in the *CBFB::MYH11* translocation) in MSCV-based retroviral vectors, transduced murine HSPCs, and performed immunofluorescence studies 4–7 days after transduction. Consistent with the TurboID protein interactome data, CBFB-GFP was predominantly nuclear, where it colocalized with RUNX1 (Figure 4). In contrast, CBFB::MYH11-GFP was predominantly localized in cytoplasmic aggregates, and endogenous RUNX1 was likewise present in these aggregates (Figure 4 and Supplemental Video 1). GFP-MYH11 was found in large aggregates that were predominantly cytoplasmic and did not contain RUNX1. In sum, these data suggest that the MYH11 domain of the fusion protein mediates cytoplasmic localization and aggregation, while the CBFB domain mediates heterodimerization with RUNX-family members.

### CBFB::MYH11 is primarily cytoplasmic in human AML cells.

**Figure 5:**
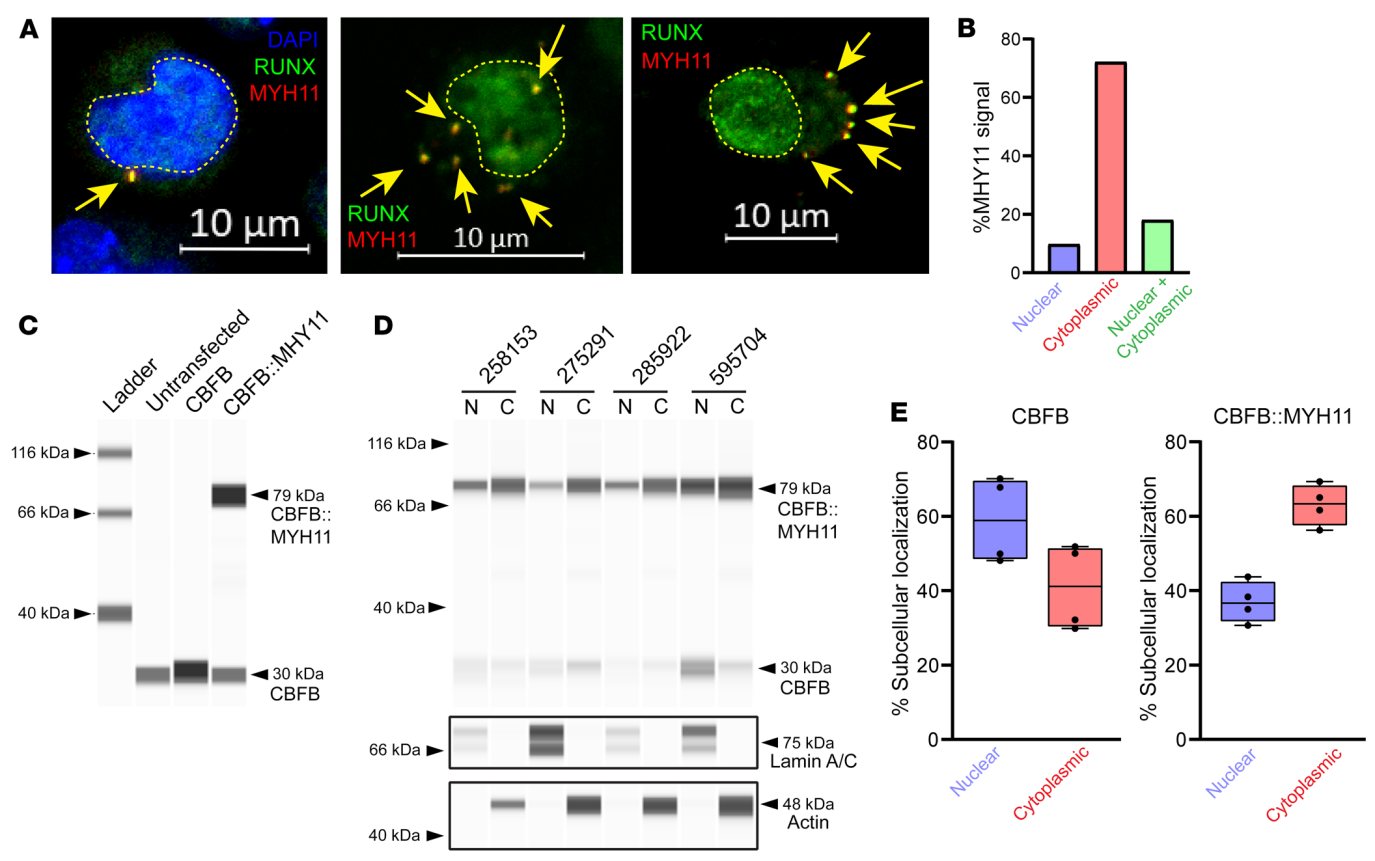
CBFB::MYH11 is predominantly cytoplasmic in human AML. (**A**) Primary human *CBFB::MYH11* AML immunofluorescence. RUNX1/2/3 is shown in green, MYH11 in red, and overlap in yellow. Yellow dashed lines outline nuclei. In a subset of images, DAPI costaining is shown in blue. Note cytoplasmic MYH11 aggregates with colocalized RUNX1 (yellow arrows) (representative images from 8 different CBFB::MYH11 patients). Scale bars: 10 μm. (**B**) Quantification of cells with nuclear-only MYH11, cytoplasmic-only MYH11, or both nuclear and cytoplasmic MYH11 (total of 338 cells scored). (**C**) K562 cells were transfected with a plasmid encoding CBFB or CBFB::MYH11, and Western blotting was performed on protein lysates using an anti-CBFB antibody. Note detection of an approximately 30 kDa band corresponding to CBFB in all lanes but increased with CBFB transfection, and detection of an approximately 79 kDa band corresponding to CBFB::MYH11 only in cells transduced with a CBFB::MYH11 plasmid. (**D**) ProteinSimple Jess blot on 4 human CBFB::MYH11 AML samples. Equal volumes of nuclear and cytoplasmic lysates were loaded. Anti–lamin A/C and anti-actin antibodies were used to verify nuclear and cytoplasmic purity. N, nuclear fraction; C, cytoplasmic fraction. (**E**) The percentage of CBFB (left panel) or CBFB::MYH11 (right panel) in the nuclear or cytoplasmic fractions of the samples shown in **D**. Each point represents an individual sample, bar indicates mean, box indicates 95% confidence interval, and whiskers indicate value range.

To validate these findings in primary human AML cells, we performed immunofluorescence studies using an antibody directed against the C-terminus of MYH11 (not detected in normal hematopoietic cells, and therefore specific for CBFB::MYH11) and an antibody that detects RUNX1, 2, and 3. While MYH11 was undetectable in non-*CBFB::MYH11* AMLs (Supplemental Figure 2), MYH11 was primarily detected in cytoplasmic aggregates in *CBFB::MYH11* AMLs, where it colocalized with RUNX proteins (Figure 5, A and B, and Supplemental Figures 2 and 3). As expected, RUNX protein was also detected in the nucleus of primary AML cells, where it may be heterodimerizing with CBFB expressed from the residual WT *CBFB* allele. In a small fraction of cells, MYH11 aggregates appeared to be nuclear; this may be either an artifact caused by “flattening” of the cells with cytospin preparations, or true nuclear CBFB::MYH11 localization in some AML cells. To address this issue, we created a 3D reconstruction of murine hematopoietic cells by spinning cells onto coverslips and allowing them to recover their normal shapes overnight to avoid flattening artifacts (Supplemental Video 1). These data suggest that “nuclear” CBFB::MYH11 in 2D representations may in fact sometimes be extranuclear.

To extend these findings, we performed nuclear-cytoplasmic fractionation in primary human *CBFB::MYH11* AMLs. Fractionation was performed using a filter-based lysis followed by centrifugation to pellet intact nuclei. Western blotting of proteins from nuclear and cytoplasmic fractions was performed using an anti-CBFB antibody that detects both WT CBFB and CBFB::MYH11 (Figure 5, C and D), an anti–lamin A/C antibody to verify nuclear enrichment (Figure 5D), and an anti-actin antibody to verify cytoplasmic enrichment (Figure 5D). Consistent with prior reports that CBFB can be found in both nuclear and cytoplasmic compartments (60, 70–73), we found that CBFB was predominantly localized in the nucleus (59.0% nuclear, range 48.1%–70.2%; Figure 5E, left panel), but also was present in the cytoplasmic fraction. In contrast, CBFB::MYH11 was predominantly cytoplasmic (63.1% cytoplasmic, range 56.3%–69.3%; Figure 5E, right panel). CBFB::MYH11 was previously reported to be found in the nuclear fraction of transfected tissue culture cells due to aggregation and buffer-induced precipitation during sample preparation (60); this may have caused an overestimate of nuclear CBFB::MYH11 abundance in this study as well. Our immunofluorescence studies of primary AML cells indeed suggest that most CBFB::MYH11 is found in cytoplasmic aggregates (Figure 5, A and B, and Supplemental Figures 2 and 3).

### CBFB N104 mediates interactions of CBFB with RUNX proteins in vitro and in vivo.

**Figure 6:**
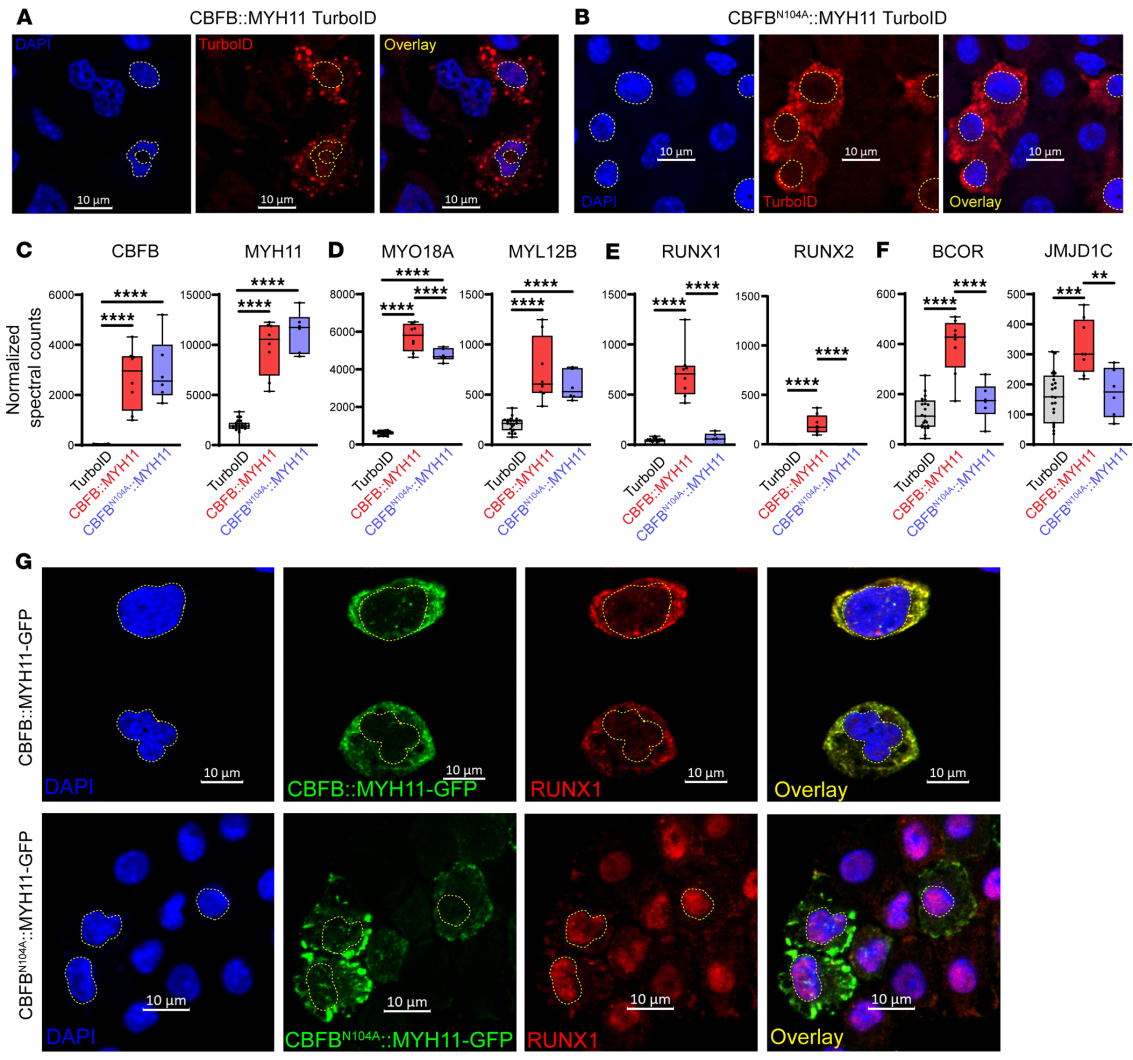
The CBFB N104 residue mediates CBFB interaction with RUNX proteins. (**A**) Immunofluorescence of CBFB::MYH11-TurboID–transduced HSPCs (**A**) or CBFB^N104A^::MYH11-TurboID–transduced HSPCs (**B**) demonstrates cytoplasmic localization of each. DAPI staining (blue) identifies nuclei, which are outlined with yellow dotted lines. TurboID is detected in red. Images are representative of 2 experiments. Scale bars: 10 μm. (**C**) Normalized spectral counts of CBFB and MYH11 in protein lysates from CBFB::MYH11-TurboID (*n* = 8) and CBFB^N104A^::MYH11-TurboID (*n* = 6) are not significantly different. One-way ANOVA between all samples, ****P < 0.0001, nonsignificant comparisons not labeled. Each point represents an individual sample, bar indicates mean, box indicates 95% confidence interval, and whiskers indicate value range. (**D**) Interactions between myosin-related proteins are maintained in CBFB^N104A^::MYH11-TurboID samples. One-way ANOVA between all samples, ****P < 0.0001, nonsignificant comparisons not labeled. (**E**) CBFB^N104A^::MYH11 disrupts the interaction with RUNX1. One-way ANOVA between all samples, ****P < 0.0001, nonsignificant comparisons not labeled. (**F**) CBFB^N104A^::MYH11 disrupts interactions between CBFB::MYH11 and other nuclear proteins, suggesting that these interactions are also mediated by RUNX-CBFB binding. One-way ANOVA between all samples, **P < 0.01, ***P < 0.001, ****P < 0.0001, nonsignificant comparisons not labeled. (**G**) Primary murine hematopoietic cells were transduced with retroviruses encoding *CBFB::MYH11-GFP* (top) or *CBFB^N104A^::MYH11-GFP* (bottom). Note that while CBFB::MYH11-GFP and CBFB^N104A^::MYH11-GFP are both cytoplasmic, RUNX1 relocalizes to the nucleus in cells expressing CBFB^N104A^::MYH11-GFP. Images are representative of 2–4 experiments. Scale bars: 10 μm.

The N104 residue of CBFB is critical for mediating the interaction of CBFB with RUNX-family proteins, and CBFB^N104A^ has been shown to abrogate the CBFB-RUNX interaction biochemically, while maintaining normal protein folding (29). We therefore generated a version of *CBFB::MYH11* carrying the *N104A* mutation fused to *TurboID* (*CBFB^N104A^::MYH11-TurboID*) and expressed this construct in primary murine hematopoietic cells, exactly as described in Figure 1A. We confirmed the cytoplasmic localization of both CBFB::MYH11-TurboID and CBFB^N104A^::MYH11-TurboID in murine hematopoietic cells, using an anti-TurboID antibody (Figure 6, A and B); as controls, we verified immunofluorescence for RUNX1::RUNX1T1-TurboID in the nucleus and PML::RARA-TurboID in nuclear microspeckles (Supplemental Figure 4), as predicted from the proximity labeling studies using the same constructs. CBFB and MYH11 were detected at similar levels in CBFB::MYH11- and CBFB^N104A^::MYH11-TurboID–transduced murine hematopoietic cells (Figure 6C). CBFB::MYH11 and CBFB^N104A^::MYH11 both interacted with myosin-related proteins (Figure 6D); however, the N104A mutation disrupted interactions with RUNX1 and RUNX2 (Figure 6E). CBFB^N104A^::MYH11 also eliminated the weak interactions of CBFB::MYH11 with BCOR and JMJD1C (Figure 6F), suggesting that these interactions were indirect, and probably mediated by RUNX1. We also expressed *CBFB^N104A^::MYH11-GFP* in murine HSPCs and performed immunofluorescence to assess localization. CBFB^N104A^::MYH11 was localized in cytoplasmic aggregates (Figure 6G, bottom, and Supplemental Video 2), similarly to CBFB::MYH11 (Figure 4; Figure 6G, top; and Supplemental Video 1). However, the N104A mutation allowed RUNX1 to return to its nuclear location, suggesting that CBFB::MYH11 aggregation and cytoplasmic localization are driven by the MYH11 domain, while RUNX1 cytoplasmic mislocalization is driven by direct interactions between the CBFB domain and RUNX1.

### RUNX gene expression is dysregulated in CBFB::MYH11 AML cells.

**Figure 7:**
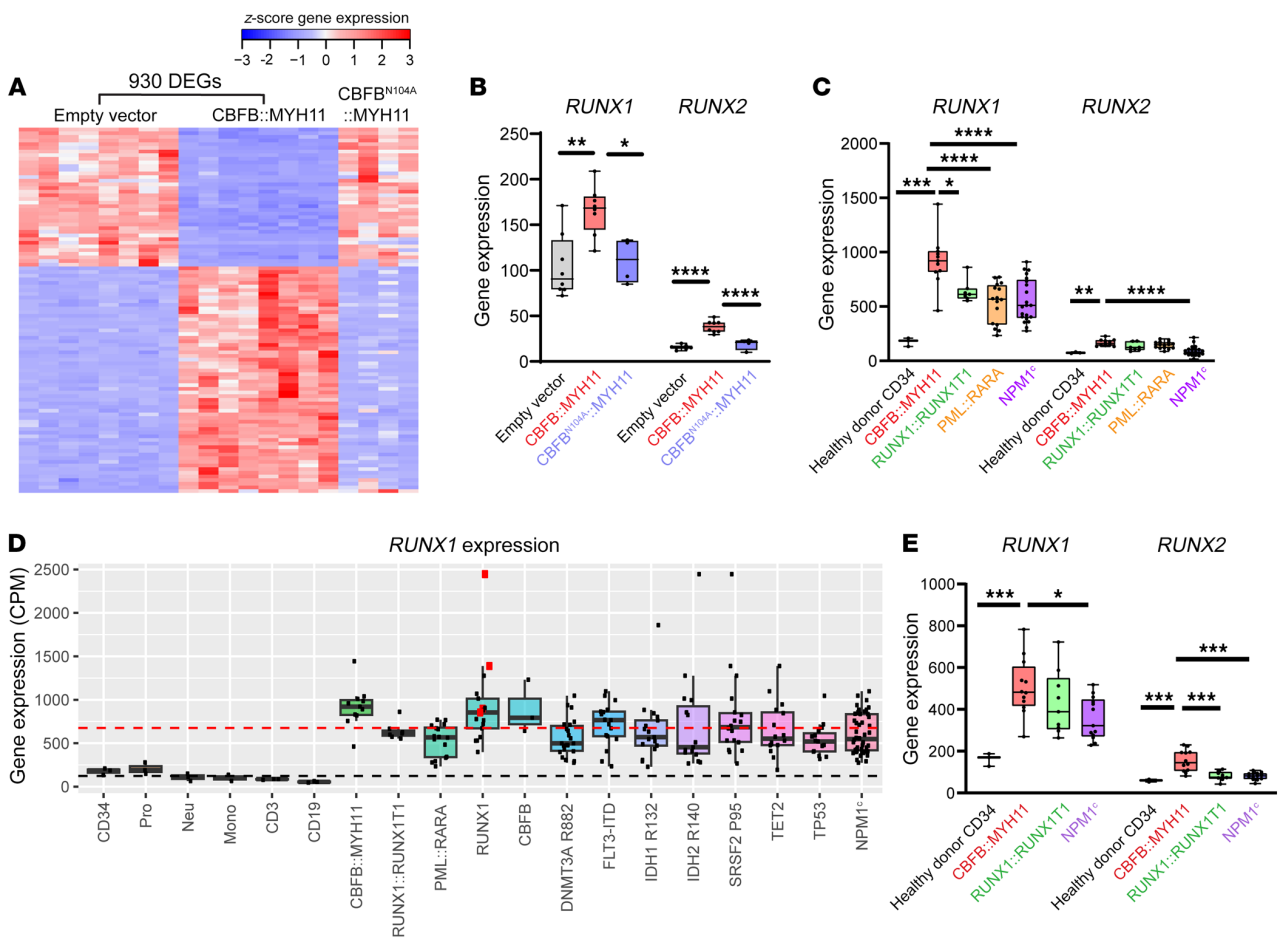
RUNX1 is dysregulated in CBFB::MYH11 AML. (**A**) Empty vector (EV; *n* = 8), *CBFB::MYH11* (*n* = 8), or *CBFB^N104A^::MYH11* (*n* = 4) was retrovirally expressed in murine hematopoietic cells, and RNA-Seq was performed at days 4 and 7. A heatmap of the top 100 DEGs shows that the *CBFB::MYH11* transcriptional signature is abrogated by the *CBFB^N104A^* mutation. (**B**) *Runx1* and *Runx2* are upregulated in *CBFB::MYH11* (red) but not *CBFB^N104A^::MYH11* (blue) cells relative to EV cells (gray), suggesting that upregulation may be due to a CBF-sensing feedback loop. One-way ANOVA between all samples, *P < 0.05, **P < 0.01, ****P < 0.0001, nonsignificant comparisons unlabeled. Each point represents an individual sample, bar indicates mean, box indicates 95% confidence interval, whiskers indicate value range. (**C**) Human TCGA AML *RUNX1/2* RNA-Seq data for healthy donor CD34^+^ (black, *n* = 3) and *CBFB::MYH11* (red, *n* = 11), *RUNX1::RUNX1T1* (green, *n* = 7), *PML::RARA* (yellow, *n* = 16), or *NPM1^c^*-mutant AMLs (purple, *n* = 21). *RUNX1* was overexpressed in *CBFB::MYH11* samples relative to all other subgroups; *RUNX2* was overexpressed relative to CD34^+^ and *NPM1^c^* subgroups. One-way ANOVA between all subgroups, *P < 0.05, **P < 0.01, ***P < 0.001, ****P < 0.0001, nonsignificant/non-*CBFB::MYH11* comparisons not shown. (**D**) Human TCGA AML *RUNX1* RNA-Seq data for healthy donor (CD34^+^; Pro, promyelocytes; Neu, neutrophils; Mono, monocytes; CD3, T cells; CD19, B cells) or AML samples with the indicated oncofusion/mutation. Red squares, biallelic *RUNX1* mutations; black dashed line, mean healthy donor cell *RUNX1* expression; red dashed line, mean AML *RUNX1* expression. All AML samples have upregulated *RUNX1* relative to healthy donor cells; AML samples with *CBFB::MYH11*, *RUNX1*, or *CBFB* mutations have mean *RUNX1* expression above the AML average. (**E**) RNA-Seq data for *RUNX1/2* from an independent cohort of healthy donor CD34^+^ (black, *n* = 3) and *CBFB::MYH11* (*n* = 12, red), *RUNX1::RUNX1T1* (*n* = 9, green), and *NPM1^c^* AMLs (*n* = 13, purple), confirming higher levels of *RUNX1/2* expression in *CBFB::MYH11* AMLs. One-way ANOVA between all subgroups, *P < 0.05, ***P < 0.001, nonsignificant/non-*CBFB::MYH11* comparisons not shown.

Feedback loops have previously been reported to regulate CBF transcriptional activity; these loops are context specific, and can include both positive and negative feedback pathways (74–79). Cytoplasmic sequestration of RUNX-family proteins by CBFB::MYH11 would be expected to decrease nuclear CBF transcriptional activity, potentially altering the transcription of *RUNX*-family members via a feedback loop. To test this hypothesis, we overexpressed *CBFB::MYH11*, *CBFB^N104A^::MYH11*, or an empty vector (EV) control in murine HSPCs using MSCV-based retroviruses, and performed RNA-Seq at days 4 and 7 after transduction. Hematopoietic cells expressing *CBFB::MYH11* had a distinct transcriptional profile relative to cells transduced with EV (Figure 7A), with more than 900 differentially expressed genes (DEGs) detected (Supplemental Table 7). In contrast, cells transduced with *CBFB^N104A^::MYH11* had an expression profile similar to that of EV-transduced cells, suggesting that the *CBFB::MYH11* transcriptional signature requires RUNX interactions. *Runx1* and *Runx2* were overexpressed relative to EV-transduced cells (Figure 7B, gray and red boxes). This overexpression was dependent on the interaction of RUNX proteins with CBFB::MYH11, since expression of *CBFB^N104A^::MYH11* did not result in compensatory upregulation of *Runx1* or *Runx2* (Figure 7B, blue boxes). These data are consistent with a model in which sequestration of RUNX proteins activates a feedback loop: *Runx1* and *Runx2* transcription may be upregulated in an attempt to compensate for reduced CBF activity.

We extended these findings to human AML cells by analyzing RNA-Seq data from The Cancer Genome Atlas (TCGA) AML study (43). We initially restricted our analysis to *CBFB::MYH11*, *RUNX1::RUNX1T1*, *PML::RARA*, and *NPM1^c^*-mutated AML (without *FLT3*-ITD mutations), all of which are favorable-risk, as well as healthy donor CD34^+^ cell controls. Consistent with the murine data, *RUNX1* was significantly overexpressed in *CBFB::MYH11* AMLs (mean expression 981.4 ± 236.1) relative to CD34^+^ HSPCs (175.9 ± 39.06), *RUNX1::RUNX1T1* AMLs (641.7 ± 103.3), *PML::RARA* acute promyelocytic leukemias (APLs) (532.0 ± 197.6), or *NPM1^c^*-mutant AMLs (553.5 ± 197.6) (Figure 7C, left). *RUNX2* was significantly overexpressed in *CBFB::MYH11* AMLs (166.4 ± 37.8) relative to CD34^+^ cells (75.5 ± 6.3) or *NPM1^c^*-mutant AMLs (91.2 ± 45.0) (Figure 7C, right). Interestingly, *RUNX1* expression in each of these AMLs was elevated in comparison with healthy donor CD34^+^ cells. We therefore extended our study to examine *RUNX1* expression in other subtypes of AML in the TCGA cohort, and found that *RUNX1* mRNA was more abundant in all AMLs tested (Figure 7D; black dashed line represents mean expression in healthy donor cells, red dashed line represents mean expression in AML samples). Consistent with a feedback loop in which *RUNX1* is upregulated in response to decreased CBF activity, the highest levels of *RUNX1* expression were detected in *CBFB::MYH11* AML, and other cases with loss-of-function mutations in either *RUNX1* (923.0 ± 471.5; Figure 7D; red squares indicate biallelic mutations) or *CBFB* (889.0 ± 305.5). *RUNX2* levels were similarly elevated in all AMLs relative to healthy donor CD34^+^ cells, with the highest expression level in *CBFB::MYH11* and biallelic *RUNX1*-mutated cases (Supplemental Figure 5). We extended our analysis of *RUNX1* and *RUNX2* expression using a recently generated RNA-Seq data set from our laboratory (80, 81), with 2 × 151 bp reads and more than 5 times the coverage of the TCGA data. This data set included healthy donor CD34^+^ samples and *CBFB::MYH11*, *RUNX1::RUNX1T1*, and *NPM1^c^*-mutant AMLs. *RUNX1* was expressed more highly in *CBFB::MYH11* AMLs (506.8 ± 136.6) relative to healthy donor CD34^+^ cells (162.3 ± 31.1) and *NPM1^c^*-mutated AMLs (356.2 ± 99.7; Figure 7E, left), and *RUNX2* was significantly overexpressed in *CBFB::MYH11* AMLs (154.4 ± 51.6) relative to healthy donor CD34^+^ cells (59.5 ± 6.7), *RUNX1::RUNX1T1* AMLs (81.1 ± 21.7), and *NPM1^c^*-mutated AMLs (80.9 ± 17.3).

The transcriptional repressor hypothesis suggests that CBFB::MYH11 interacts with transcriptional repressors in the nucleus via the MYH11 “tail,” recruiting transcriptional repression machinery to CBF DNA binding sites in a mechanism similar to that of RUNX1::RUNX1T1. This hypothesis suggests that *CBFB::MYH11* and *RUNX1::RUNX1T1* AMLs should have similar transcriptional profiles. Consistent with previously published data (43, 46), we found that *CBFB::MYH11* and *RUNX1::RUNX1T1* AMLs had distinct transcriptional signatures (Supplemental Figure 6A). While some DEGs relative to healthy donor CD34^+^ cells were shared between the 2 AMLs, most DEGs were also shared with *NPM1^c^-*mutated AML, suggesting that they may represent AML-specific dysregulated genes, rather than a CBF-specific AML transcriptional signature (Supplemental Figure 6, B and C, and Supplemental Tables 8–11).

### The RUNX1/CBFB expression ratio is altered in human AML samples.

**Figure 8:**
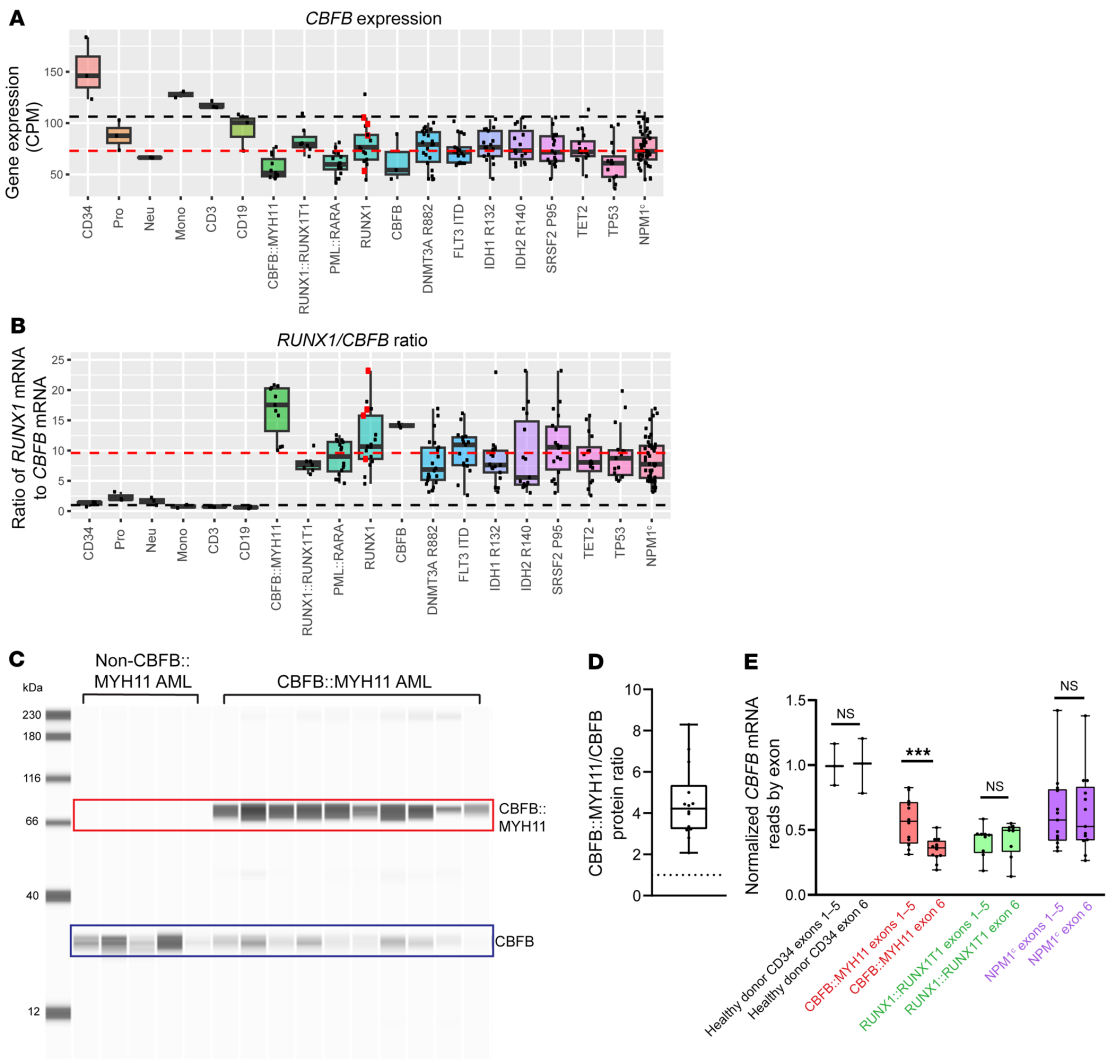
*RUNX1/CBFB* expression ratio is disrupted in human AML. (**A**) TCGA LAML *CBFB* RNA-Seq data for the indicated healthy donor cells or AMLs. Black dashed line, mean healthy donor expression; red dashed line, mean AML expression. *CBFB* mRNA levels are lower in all AMLs, relative to healthy donor CD34^+^ cells. Each point represents an individual sample, bar indicates mean, box indicates 95% confidence interval, whiskers indicate value range. (**B**) Ratio of normalized, length-scaled *RUNX1/CBFB* mRNA expression. Black dashed line, 1:1 ratio; red dashed line, mean AML ratio. All AMLs have elevated *RUNX1/CBFB* ratios relative to healthy donor samples; *CBFB::MYH11*, *RUNX1*, and *CBFB*-mutated AMLs have the highest ratios. (**C**) Representative Jess blot (total of 7 experiments) of human non-*CBFB::MYH11* (*n* = 6 patients) or *CBFB::MYH11* (*n* = 14 patients) AML protein lysates for CBFB. Upper band (red box) indicates CBFB::MYH11; lower band (blue box) indicates WT CBFB. (**D**) CBFB::MYH11 to CBFB ratio in CBFB::MYH11 AMLs (*n* = 14 patients). Each point represents 1 patient; for patients in whom a sample was assayed more than once, point indicates mean of all assays. Dotted line, 1:1 ratio. The average CBFB::MYH11/CBFB ratio was 4.5:1, indicating that CBFB::MYH11 protein is more abundant than CBFB protein in primary human AMLs. (**E**) *CBFB* mRNA read counts from the validation cohort (Figure 7E) normalized to healthy donor CD34^+^ expression mean, grouped by exons 1–5 (unaffected by *CBFB::MYH11* translocation) or exon 6 (lost with *CBFB::MYH11* translocation). *CBFB* exons 1–5 expression is similar in *CBFB::MYH11*, *RUNX1::RUNX1T1*, and *NPM1^c^* AMLs, suggesting that *CBFB* locus transcriptional activity is unaffected by *CBFB::MYH11* translocation. *CBFB* exon 6 reads are decreased by approximately 50% relative to exons 1–5 in *CBFB::MYH11* AML, consistent with translocation-induced loss of one *CBFB* exon 6 allele. Paired *2*-tailed *t* test between exons 1–5 and exon 6 reads within each group, ***q < 0.001.

We next examined *CBFB* gene expression in the TCGA data set. *CBFB* expression was decreased in all AMLs relative to healthy donor CD34^+^ cells (Figure 8A). We then calculated the *RUNX1* to *CBFB* mRNA expression ratio for all samples. While healthy donor CD34^+^ cells, promyelocytes, neutrophils, monocytes, T cells, and B cells all had *RUNX1/CBFB* mRNA expression ratios of approximately 1:1 (Figure 8B, black dashed line; CD34^+^ ratio 1.2:1 ± 0.4), consistent with the 1:1 RUNX/CBFB required for CBF activity, all tested AML samples had elevated *RUNX1*/*CBFB* ratios (Figure 8B; red dashed line indicates AML mean ratio of 9.6:1 ± 4.8). Consistent with their elevated levels of *RUNX1*, the highest *RUNX1/CBFB* ratios were in *CBFB::MYH11* AMLs (16.6:1 ± 4.3), *RUNX1*-mutated AMLs (all *RUNX1*-mutant AMLs, 11.8:1 ± 4.9; biallelic *RUNX1*-mutant in red squares, 16.1 ± 6.0), and *CBFB*-mutated AMLs (14.2:1 ± 0.4).

Since *CBFB::MYH11* is transcribed from the *CBFB* locus in AML cells, the amount of CBFB::MYH11 protein relative to WT CBFB protein has been presumed to be equivalent. If CBFB::MYH11 sequesters RUNX1 from WT CBFB, relative protein abundance may be an important factor in determining the size of the sequestration effect. We therefore determined the relative ratio of CBFB::MYH11 to CBFB protein in primary human AML samples (14 from *CBFB::MYH11* patients and 6 from patients without this fusion). We prepared protein lysates from samples cryopreserved at presentation, and performed Western blot analysis using a CBFB-specific antibody (Figure 5C). Since the antibody detects the same epitope in both WT CBFB and CBFB::MYH11, the Western blot signal directly corresponds to relative protein abundance. In all samples, we detected an approximately 29 kDa band representing WT CBFB (Figure 8C, blue box). The upper band of approximately 79 kDa was only detected in *CBFB::MYH11* AML samples, representing the CBFB::MYH11 fusion protein (Figure 8C, red box). If CBFB::MYH11 and CBFB were equally abundant, the protein ratio would be 1:1 (Figure 8D, black dotted line). Instead, the mean protein ratio was 4.5:1 ± 1.7 across all tested cases (*n* = 14). Since patient samples inevitably contain a mix of leukemic and nonleukemic cells (Supplemental Table 12), and contaminating nonleukemic cells contain CBFB but not CBFB::MYH11, the ratio of 4.5:1 may underestimate the true ratio in AML cells.

To explore whether the increased abundance of CBFB::MYH11 was due to a transcriptional or post-transcriptional effect, we examined total RNA-Seq data from our deep-coverage RNA-Seq cohort. WT *CBFB* contains 6 exons, and the *CBFB* breakpoint in the *CBFB::MYH11* translocation occurs at the 3′ end of *CBFB* exon 5. WT *CBFB* therefore contains *CBFB* exons 1–6, while *CBFB::MYH11* only contains *CBFB* exons 1–5. *CBFB::MYH11* expression is presumed to be controlled by regulatory regions in the *CBFB* locus. We found no evidence for increased abundance of *CBFB* exons 1–5 in *CBFB::MYH11* AMLs, relative to *RUNX1::RUNX1T1* or *NPM1^c^*-mutated AMLs (Figure 8E), suggesting that the transcriptional activity of the *CBFB* locus is not substantially altered by the fusion in primary AML cells. As expected, exon 6 transcript levels were reduced by approximately 50% in *CBFB::MYH11* AMLs relative to exons 1–5, consistent with replacement of one *CBFB* exon 6 allele with *MYH11* due to the translocation. Combined, these data suggest that the CBFB::MYH11 fusion protein may have a prolonged half-life compared with CBFB; this would increase approximately 4.5-fold the amount of RUNX1 that could be sequestered by the fusion protein. Interestingly, several ubiquitination-related proteins had reduced interactions with CBFB::MYH11-TurboID relative to CBFB-TurboID (Figure 3E and Supplemental Figure 7), suggesting that decreased interactions with ubiquitin ligases may contribute to prolonged CBFB::MYH11 half-life.

## Discussion

Favorable-risk AML oncofusion proteins have been extensively studied. Our report builds on this important body of work in several ways: (a) we used proximity labeling and unbiased proteomic approaches to demonstrate fusion oncoprotein interactions in primary hematopoietic cells, rather than transformed cell lines; (b) we used modern imaging technology and primary hematopoietic cells, rather than nonhematopoietic cells or cell lines, to evaluate CBFB::MYH11 intracellular localization; (c) we validated CBFB::MYH11 cytoplasmic localization in unmanipulated primary human AML samples, using immunofluorescence, and nuclear-cytoplasmic fractionation with Western blotting; (d) we demonstrated that CBFB::MYH11 protein is approximately 4.5 times more abundant than WT CBFB in primary human AML cells, perhaps owing to a prolonged half-life; (e) we utilized the CBFB^N104A^ mutation (which does not disrupt CBFB folding) to define RUNX interaction–dependent effects, including RUNX1 cytoplasmic sequestration, the *CBFB::MYH11* transcriptional program, and *Runx1/2* upregulation; (f) we identified multiple myosin-related interacting proteins that may explain CBFB::MYH11’s cytoplasmic localization; and (g) we demonstrated that increased expression of *RUNX1* — and reduced expression of CBFB — are canonical features of AML cells, suggesting a generalized role in AML pathogenesis.

A transcriptional repressor role for CBFB::MYH11 has been postulated by multiple groups, based on coimmunoprecipitation experiments (39–42, 82–84) summarized in Supplemental Table 13. Interactions with HDAC8 and SIN3A (39, 42), but not HDAC1 (40), have been reported to require the MYH11 C-terminus. However, fusion of the MYH11 C-terminus to RUNX1, which would be expected to recruit transcriptional repressor machinery to the same loci, did not phenocopy CBFB::MYH11 (82), suggesting that CBFB::MYH11 does not function solely by recruiting repressors to CBF target genes. Furthermore, in assays performed in cell lines, CBFB::MYH11 impairs recruitment of RUNX1 protein to a target promoter, rather than recruiting transcriptional repressors (45, 82). The transcriptional repressor model for CBFB::MYH11 is also complicated by the recent discovery of a recurrent *CBFB^D87GDSY^* insertional mutation in a subset of AMLs that are transcriptionally similar to *CBFB::MYH11* AML (85), without the presence of any domains predicted to interact with transcriptional repressors.

We did not see strong evidence for CBFB::MYH11 interactions with transcriptional repressor machinery using the proximity labeling system; CBFB::MYH11-TurboID only weakly interacted with BCOR, and this interaction was lost with CBFB^N104A^::MYH11, suggesting that the interaction was probably mediated indirectly by RUNX1. Our experimental design differs from previous studies in several important ways. First, many of these studies used nonhematopoietic cells or cell lines, which have distinct biology and protein contexts from the primary hematopoietic cells used here. Second, our study used labeling in live cells with intact subcellular compartmentalization under physiologic salt and osmotic conditions, with stringent wash steps designed to reduce nonspecific interactions. Disruption of intracellular compartments prior to identification of interacting proteins may permit nonphysiologic interactions to occur, and the sensitivity of these interactions to buffer conditions has been well described, including in some of the experiments described above (39, 42). Finally, the TurboID system requires only proximity, not direct interaction, and it is therefore more permissive than systems requiring direct protein-protein interaction. Clearly, TurboID fusion proteins are able to enter the nucleus and detect transcriptional repressor interactions, as shown by the *RUNX1::RUNX1T1* and *PML::RARA* data (Figure 2 and Supplemental Figure 4). It is unlikely that CBFB::MYH11-TurboID is excluded from the nucleus because of its size, since it is actually the smallest of the oncofusions tested in this study (Figure 1, B and C). While CBFB::MYH11 overexpression could conceivably result in the detection of nonphysiologic interactions, it would not be expected to result in an inability to detect physiologic interactions.

The subcellular localization of CBFB::MYH11 has long been controversial. Many investigators have performed experiments designed to define subcellular localization by immunofluorescence and/or nuclear-cytoplasmic fractionation and Western blotting (41, 60, 71, 72, 82, 86–91), resulting in a complex body of data with contradictory results, summarized in Supplemental Table 14. Even in settings where CBFB::MYH11 was confirmed to be cytoplasmic by immunofluorescence, nuclear-cytoplasmic fractionation produced substantial bands in the nuclear fraction, which could potentially be attributed to CBFB::MYH11 aggregates copurifying with the nuclear pellet due to buffer conditions, as suggested by Adya et al. (60). ChIP-Seq studies in cell lines (83, 84) and transformed murine leukemias (40) using CBFB and MYH11 antibodies have reported CBFB::MYH11 enrichment at specific target gene sites; however, CBFB::MYH11 binding was associated with upregulated genes (40) or both up- and downregulated genes (83, 84), which is inconsistent with the hypothesis that this fusion creates a transcriptional repressor phenocopying the mechanism of RUNX1::RUNX1T1. Although our model system has caveats associated with overexpression (i.e., high levels of CBFB::MYH11 may affect its intracellular distribution), we orthogonally validated these findings using primary human AML samples with physiologic CBFB::MYH11 expression. These samples also showed predominantly cytoplasmic CBFB::MYH11 aggregates with RUNX colocalization (Figure 5A and Supplemental Figures 2 and 3), and we confirmed that CBFB::MYH11 was primarily cytoplasmic in human AML samples using nuclear-cytoplasmic fractionation and Western blotting. Given the inherent sensitivity limits of immunofluorescence, we are unable to rule out the possibility that low-level nuclear CBFB::MYH11 may also be present in these cells. However, any model of CBFB::MYH11 pathogenesis must account for the cytoplasmic aggregates containing both CBFB::MYH11 and RUNX1 in primary human AML cells.

Our proximity labeling data suggest that the cytoplasmic localization of CBFB::MYH11 may be due to interaction of the MYH11 domain with cytoplasmic myosin-related proteins (Figure 2, D and E). When the MYH11 domain is expressed without the CBFB domain, it is indeed predominantly cytoplasmic (Figure 4). The MYH11 domain of CBFB::MYH11 retains the coiled-coil and assembly competence domains, regions of the MYH11 that mediate homo-oligomerization. This domain has been reported to be essential for the transforming activity of CBFB::MYH11 (92, 93). While prior studies suggested that the MYH11 moiety may interact with transcriptional repressors in the nucleus (39, 40, 42), we did not detect these interactions with proximity labeling. Others have suggested that CBFB::MYH11 may bind to cytoskeletal actin filaments (60), or that CBFB itself may bind to the actin-binding protein filamin A (FLNA) (73). Our TurboID studies did not show any evidence for specific CBFB-FLNA or CBFB::MYH11-actin interactions, consistent with the fact that *CBFB::MYH11* rearrangements exclude the actin-binding domain of MYH11. More likely, the MYH11 domain hetero-oligomerizes with other myosin-related proteins, perhaps as a result of homology to these proteins in the coiled-coil and assembly competence domains.

All major models of the CBFB::MYH11 mechanism have postulated reduced activity of core binding factor target genes. A constitutive *CBFB::MYH11*-knockin model phenocopied *Cbfb* or *Runx1* knockouts (94), with embryonic lethality due to hematopoietic failure (22, 95–98). Further, *CBFB::MYH11* expression has been shown to reduce CBF transcriptional activity in multiple cell lines (39, 45, 86, 87). Some level of residual RUNX activity appears to be required, however, since deletion of *Runx1* eliminates the ability of *CBFB::MYH11* to induce AML (99, 100), while overexpression of *Runx2* accelerates leukemogenesis (101, 102). Preclinical models using small-molecule inhibitors impairing the CBFB::MYH11 interaction with RUNX proteins also show the key role this interaction plays in maintaining transformed AML cells (103–108). The importance of RUNX1 is recapitulated in our experiments, since the transcriptional signature of *CBFB::MYH11* expression in murine hematopoietic cells is eliminated by the *CBFB^N104A^::MYH11* mutation, which mitigates the interaction of this protein with RUNX proteins (29). CBFB::MYH11-mediated overexpression of *Runx1/2* in murine hematopoietic cells is consistent with a model of compensatory upregulation due to decreased CBF activity. We detected similar findings in primary human AML samples, with upregulation of *RUNX1* and downregulation of *CBFB* in all 176 AMLs tested. The samples with the most dramatically dysregulated *RUNX1/CBFB* ratio had *CBFB::MYH11*, *RUNX1*, or *CBFB* loss-of-function mutations, which are all predicted to cause severe reductions in CBF activity. Increased *RUNX1* mRNA levels in *RUNX1*-mutated AML samples might appear to be paradoxical, since some *RUNX1* mutations cause frame shifts and/or premature stop codons that might be expected to cause nonsense-mediated decay. However, manual review of *RUNX1* transcripts in all TCGA AMLs with *RUNX1* mutations revealed no evidence of truncated mRNA (data not shown).

We do not yet know whether increased *RUNX1* mRNA in AML samples leads to an increase in the abundance of “uncomplexed” RUNX1 protein. RUNX1 is the DNA-binding component of CBF, and its affinity for target DNA binding is increased more than 40-fold by CBFB heterodimerization (25). Excess uncomplexed RUNX1 protein would be expected to be minimally active in AML cells. Further, CBFB has been shown to stabilize RUNX1 protein in cell lines and *Cbfb^–/–^* whole-embryo extracts (109), suggesting that uncomplexed RUNX1 protein may be rapidly ubiquitinated and degraded. In either case, the reduction of CBF activity should be magnified in *CBFB::MYH11* AML cells, which contain 4.5 times more CBFB::MYH11 than CBFB, increasing the size of the “sequestration sink” for RUNX1 in the cytoplasm. Taken together, these findings demonstrate the dysregulated stoichiometry of CBFB and RUNX1 mRNA and protein levels in nearly all AML samples, suggesting broad relevance for AML pathogenesis.

## Methods

### TurboID MSCV vector creation.

TurboID cDNA sequence was obtained from Addgene plasmid 107171. A glycine-serine linker in-frame with TurboID was synthesized by Integrated DNA Technologies and subcloned into MSCV-IRES-GFP by Genewiz/Azenta. *V5-PML::RARA*, *RUNX1::RUNX1T1*, *CBFB::MYH11*, *CBFB^N104A^::MYH11*, *CBFB* transcripts 1 and 2, *NPM1*, and *NPM1^cA^* cDNAs were cloned using conventional restriction digest cloning or Gibson assembly. Sequence was confirmed for all plasmids using Sanger sequencing of the insert, or whole-plasmid sequencing by Plasmidsaurus. N- and C-terminal TurboID cloning plasmids were deposited in Addgene (plasmid IDs 207957 and 207958).

### TurboID proximity labeling.

TurboID proximity labeling and mass spectrometry were performed as previously described (47). Briefly, bone marrow from 8- to 16-week-old WT C57BL/6J mice (The Jackson Laboratory) was extracted using standard techniques and incubated overnight in RPMI 1640 (Gibco) containing 15% FCS (R&D Systems), penicillin-streptomycin (Gibco), 100 ng/mL SCF, 10 ng/mL thrombopoietin, 50 ng/mL FLT3L, and 6 ng/mL IL-3 (cytokines from PeproTech). Lineage depletion was performed the following day using the Direct Lineage Cell Depletion Kit and the autoMACS magnetic cell separator (Miltenyi Biotec). Cells were transduced with retroviruses 1 day after lineage depletion. Retroviruses were prepared by transfection of GP2 293 cells (Takara) with TurboID target plasmids and the VSVg packaging plasmid using TransIT-LT1 (Mirus Bio) or Lipofectamine 3000 (Invitrogen). Retroviruses were adsorbed to tissue culture plates coated with RetroNectin (Takara), and cells were spun onto adsorbed retrovirus. Transductions were performed on 2 consecutive days. After 1 day of recovery, GFP^+^ cells were isolated by flow to enrich for transduced cells. Two days after sorting, 1 million to 5 million GFP^+^ sorted cells were resuspended at 1 million cells/mL in medium containing 50 μM biotin (MilliporeSigma), incubated 4 hours at 37°C, washed with PBS, and lysed in 25 mM Tris-HCl, 150 mM NaCl, 1% Triton X-100, pH 7.2, with protease inhibitor cocktail (MilliporeSigma P8340). Lysates were sonicated, then incubated with high-capacity streptavidin resin beads (Thermo Fisher Scientific) overnight at 4°C on a rotator. Beads were washed once with 1% SDS in PBS, and twice with 50 mM Na_2_HPO_4_, 500 mM NaCl, 1% Triton X-100, pH 7.4. Proteins were reduced with either DTT or TCEP, alkylated with iodoacetamide, and eluted from beads by tryptic digestion. Trapped ion mobility time-of-flight mass spectrometry was performed using a *nano-Elute* chromatograph (Bruker Daltonics) interfaced with a timsTOF Pro mass spectrometer as previously described (47); see Supplemental Methods for additional detail. Peptides were filtered in Scaffold (version 5.2.1, Proteome Software) at 1% FDR by searching against a reversed protein sequence database. A minimum of 2 peptides were required for protein identification. Spectral counts were normalized by trimmed mean of M values (TMM) using edgeR (110). A minimum of 5 spectral counts per million in each sample were required for a protein to be considered detected above background. Spectral counts of detected proteins were compared against controls using edgeR, and differentially interacting proteins (DIPs) were defined as having a more than 2-fold increase and FDR less than 0.05.

### Nuclear protein complexes.

A list of high-confidence proteins of defined nuclear complexes was manually curated based on literature review and gene ontogeny lists (Supplemental Table 2).

### Pathway enrichment.

Pathway enrichment for DIPs from the CBFB-TurboID versus CBFB::MYH11-TurboID comparison was performed using ToppFun software (https://toppgene.cchmc.org/enrichment.jsp), and curated statistically significant gene ontogeny sets were selected. FDR refers to the Benjamini-Hochberg FDR. Ratio indicates the number of DIPs detected divided by the number of genes in the gene set.

### Western blotting.

Western blots were performed using the ProteinSimple Jess system (Bio-Techne) using the following antibodies: BirA/TurboID (Novus NBP2-59939), MYO18A (ProteinTech 14611-1-AP), NCOR2 (Novus NB120-5802), CBFB (Santa Cruz Biotechnology sc-56751), lamin A/C (Cell Signaling Technology 4777), and actin (MilliporeSigma MAB1501). Relative quantification was performed using peak fit area under protein curves with Compass software (Bio-Techne). K562 cells were obtained from ATCC. For AML protein extracts, cryovials from presentation bone marrow, leukapheresis, or peripheral blood samples were thawed into 50% FBS/50% PBS; most samples were incubated with 500 mM DFP (MilliporeSigma) for 10 minutes on ice to inactivate myeloid serine proteinases to decrease protein degradation. Cells were washed with PBS 3 times, collected by centrifugation at 90*g* for 10 minutes, filtered over a 50 μm filter (Systex), counted, lysed at 1 × 10^6^ cells per 100 μL NuPAGE LDS buffer (Invitrogen), and sonicated. For nuclear-cytoplasmic fractionation, thawed, washed cells from presentation bone marrow vials were fractionated into nuclear or cytoplasmic compartments using the Minute Plasma Membrane Protein Isolation and Cell Fractionation Kit (Invent Biotechnologies SM-005). The nuclear pellet was lysed in a volume of RIPA buffer equivalent to the volume of the cytoplasmic fraction.

### Murine immunofluorescence.

*CBFB* exons 1–5, *CBFB::MYH11*, *CBFB^N104A^::MYH11*, and *MYH11* exons 33–41 were fused in-frame to GFP in MSCV vectors. Murine hematopoietic cells were transduced as described above. Four or seven days after transduction, cells were harvested and spun onto Superfrost Plus slides (Thermo Fisher Scientific). Cells were fixed for 20 minutes at room temperature with Image-iT paraformaldehyde fixative solution (Invitrogen), washed 3 times for 5 minutes with PBS, and blocked for at least 30 minutes at room temperature in 10% goat serum in PBS plus 1% BSA (MilliporeSigma) plus 0.1% saponin (MilliporeSigma). Slides were washed with PBS for 5 minutes, then incubated with primary antibody (RUNX1 antibody, Novus NBP1-89105; BirA/TurboID antibody, Novus NBP2-59939) overnight at 4°C in staining buffer (PBS, 1% BSA, 0.1% saponin). Slides were washed 3 times for 5 minutes with PBS, and incubated with secondary antibodies (anti-rabbit–Alexa Fluor 647, Invitrogen A27040, or anti-mouse–Alexa Fluor 633, Invitrogen A21052) and 1 μg/mL DAPI for at least 45 minutes at room temperature in staining buffer. Slides were washed 3 times for 5 minutes with PBS and mounted using Prolong Glass Antifade (Invitrogen). Slides were imaged using a Zeiss LSM880 laser scanning confocal microscope; a subset were imaged with Airyscan processing. Images were processed using Zen Blue software (Zeiss). Animated *Z*-stack movies were generated by the spinning of transduced cells onto retronectin-coated coverslips and incubation of cells overnight in a tissue culture incubator, followed by fixation and staining as above. The entire cell depth was imaged confocally, and videos were produced using Volocity (Quorum Technologies).

### Human immunofluorescence.

Primary presentation bone marrow, blood, or leukapheresis cryovials were thawed and washed as above. Cells were incubated at 37°C in DMEM (Gibco), 20% FBS (R&D Systems), penicillin-streptomycin, 50 nM β-mercaptoethanol (MilliporeSigma), 100 ng/mL SCF, 10 ng/mL FLT3L, 10 ng/mL TPO, 10 ng/mL IL-3, and 20 ng/mL IL-6 (cytokines from PeproTech) for 1–5 days before cytospins were made as described above. Cells were blocked and permeabilized as described above. Slides were then stained with primary MYH11 antibody (Novus NBP2-66967) overnight at 4°C, washed, and stained for at least 45 minutes with anti-rabbit–Alexa Fluor 647, as above. For cells costained for RUNX1/2/3, cells were then washed, reblocked with 10% rabbit serum in staining buffer for at least 4 hours at room temperature, washed with PBS, and incubated overnight at 4°C with Alexa Fluor 488–conjugated RUNX1/2/3 (Abcam ab199221) and 1 μg/mL DAPI. Cells were then washed, mounted, and imaged as above. Cells with visually detectable MYH11 were imaged.

### Murine RNA-Seq.

Murine hematopoietic cells were transduced with MSCV-based retroviruses containing no cDNA (“empty vector”) or cDNAs encoding *CBFB::MYH11* or *CBFB^N104A^::MYH11* (MSCV-cDNA-IRES-GFP) and harvested at days 4 and 7 after transduction. RNA was extracted using Quick-RNA Miniprep kit (Zymo). Total RNA-Seq followed by differential gene analysis using kallisto (111) and edgeR (110) was performed as previously described (112). Differentially expressed genes were defined as having a greater than 2-fold change and FDR less than 0.05.

### Human RNA-Seq.

LAML TCGA data were obtained from the Database of Genotypes and Phenotypes (dbGaP; phs000159v13.p5). Validation cohort data for healthy donor CD34^+^ cells, *NPM1^c^*-mutated AML, and *CBFB::MYH11* were obtained from previously published data sets (80, 81). *RUNX1::RUNX1T1* AML RNA-Seq was performed as previously described (80, 81). Differentially expressed genes were defined as having greater than 2-fold change and FDR less than 0.05.

### Statistics.

Pairwise comparisons of manually selected TurboID-labeled proteins or RNA transcripts were performed using 1-way ANOVA with post hoc Tukey’s testing and Prism software (GraphPad); *P* values less than 0.05 were considered significant. Unbiased differential analysis of TurboID and RNA-Seq data was performed using the edgeR package (110), requiring greater than 2-fold increase and FDR less than 0.05. The relative transcriptional abundance of *CBFB* exons 1–5 versus exon 6 was compared by paired 2-tailed *t* test with multiple-comparison testing by 2-stage step-up (Benjamini, Krieger, and Yekutieli), and *q* values less than 0.05 were considered significant.

### Study approval.

Human samples were acquired as part of studies approved by the Human Research Protection Office at Washington University. All patients provided written informed consent prior to participation under IRB protocol 201011766. Murine studies were approved under IACUC protocol 23-0041.

### Data availability.

TurboID data are publicly available in the MassIVE database (data set MSV000092615). Normalized data for detected proteins for each TurboID fusion construct are also available in an online Shinyapp at https://leylab.org/Favorable_Risk_AML_TurboID.html Murine RNA-Seq data are publicly available in the NCBI Short Read Archive (PRJNA1018524). Human RNA-Seq data are available in dbGaP (phs000159v13.p5). Normalized spectral counts for differentially interacting proteins for TurboID experiments are in Supplemental Tables 1 and 3–6. Normalized RNA-Seq reads for differentially expressed genes for human and murine data sets are in Supplemental Tables 7–10. Data represented graphically are also available in the Supporting Data Values file.

## Author contributions

RBD, JAH, CDSK, and FF performed research. RBD, ZX, SMR, CAM, and TJL analyzed data. PEG, RWS, YM, and RRT performed mass spectrometry, data processing, and proteomic analysis. RBD and TJL designed the study and wrote the manuscript.

## Supplementary Material

Supplemental data

Supplemental tables 1-12

Supplemental video 1

Supplemental video 2

Supporting data values

## Version 1 12/07/2023

In-Press Preview

## Version 2 02/15/2024

Electronic publication
