# Supplementary material for: Proteogenomic analysis reveals cytoplasmic sequestration of RUNX1 by the acute myeloid leukemia–initiating CBFB::MYH11 oncofusion protein
Source: J Clin Invest. 2023 Dec 7;134(4):e176311. doi: 10.1172/JCI176311 (PMC10866659; doi:10.1172/JCI176311)
Supplement: Supplemental data [file jci-134-176311-s177.pdf]

SUPPLEMENTARY MATERIALS

Supplementary Materials Table of Contents……………………………………………………………..1
Figure S1………………………………………………………………………………………….........2
Figure S2………………………………………………………………………………………….........3
Figure S3…………………………………………………………………………………………………4
Figure S4……………………………………………………………………………………………..…5
Figure S5…………………………………………………………………………………………………6
Figure S6………………………………………………………………………………………………..7
Figure S7…………………………………………………………………………………………………8
Captions for Tables S1-S12……………………………………………………………………………..9
Table S13………………………………………………………………………………………………...10
Table S14………………………………………………………………………………………………...11
Captions for Videos S1-S2……………………………..………………………………………………12
Supplementary methods………………………………………………………………………………..13
Supplementary references……………………………………………………………………………..14

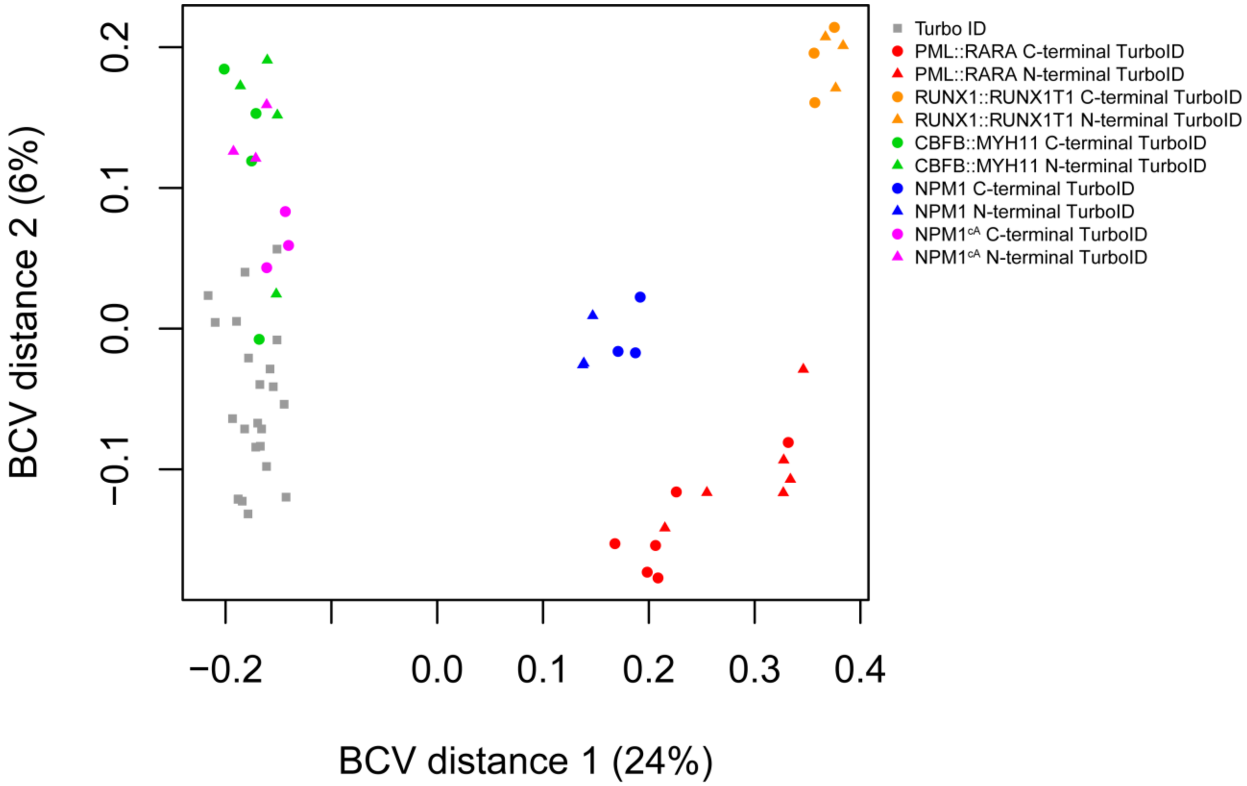

Figure S1. Multidimensional scaling plot of TurboID data with the indicated TurboID fusion proteins, separated by N- (triangles) or C-terminal (circles) TurboID fusion. N- and C-terminal fusions do not cluster independently. BCV = biological coefficient of variation.

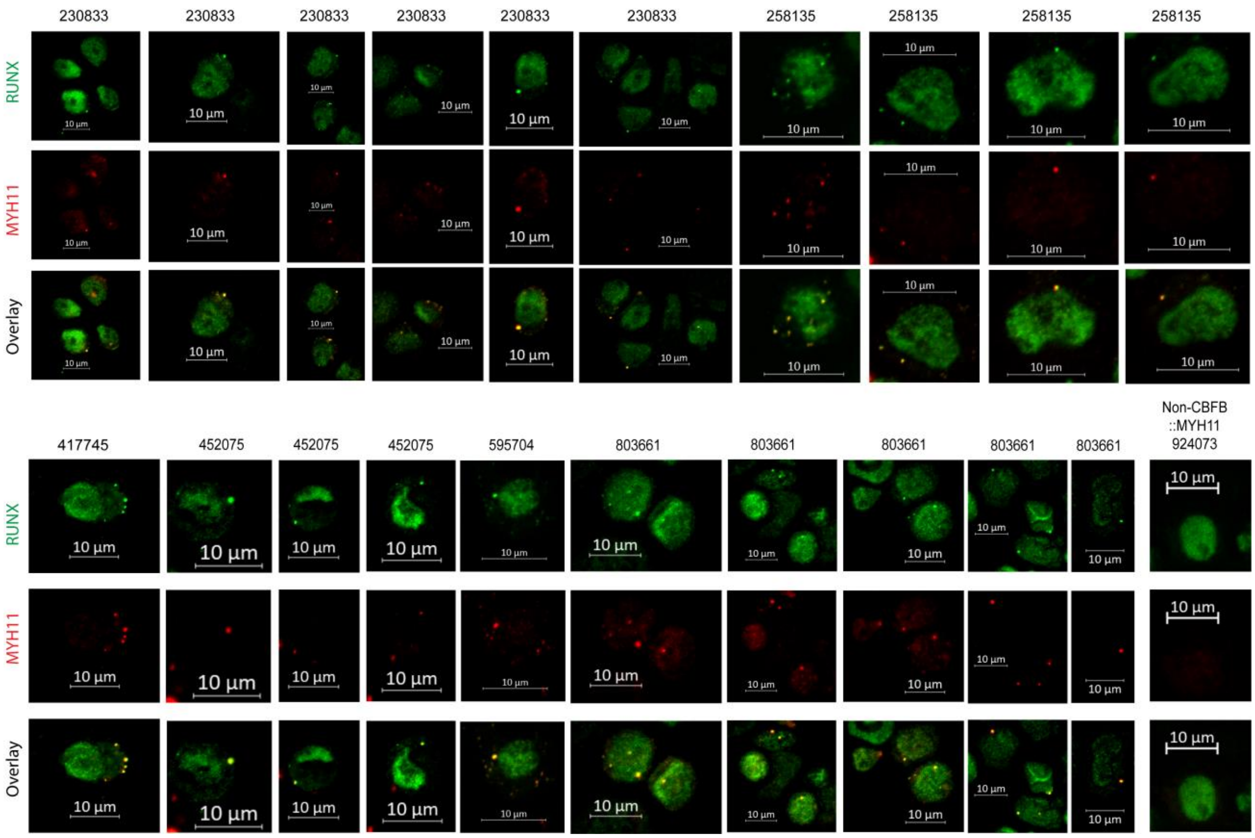

Figure S2. Primary human AML cells stained with MYH11 (red) and RUNX1/2/3 (green). Overlap is shown in yellow. The number above each image refers to the unique patient identifier. All samples are from *CBFB*::*MYH11* patients except the final sample (924073). Note cytoplasmic MYH11 aggregates with RUNX colocalization in *CBFB*::*MYH11* patient samples. Overlay images for 258135 and 417745 are repeated from **Figure 5A**.

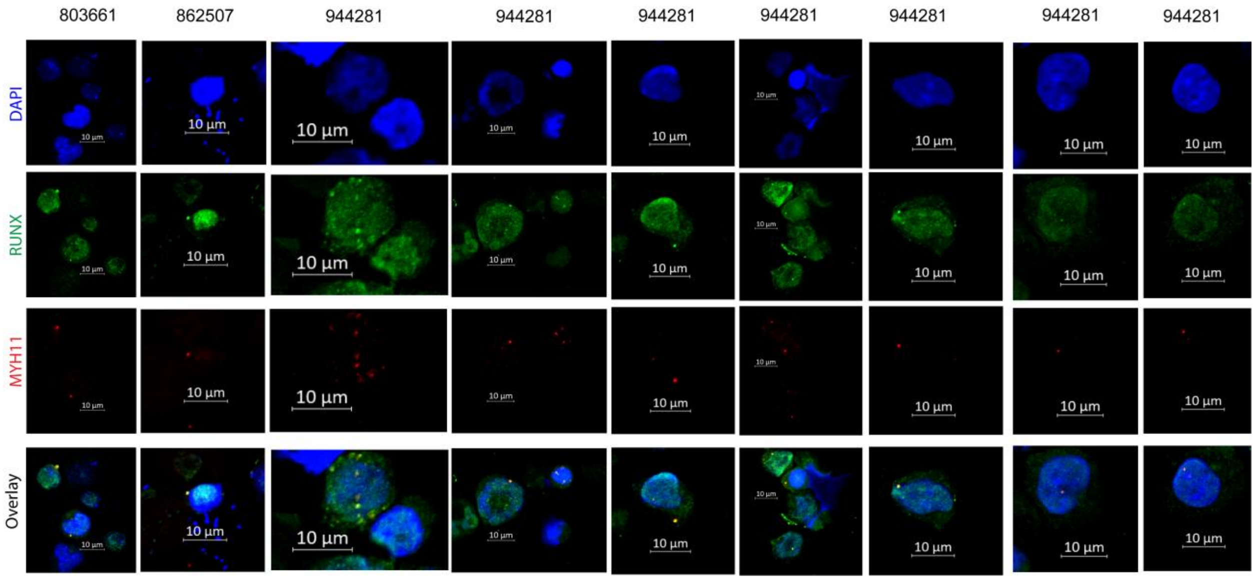

Figure S3. Primary human AML cells stained with DAPI (blue), MYH11 (red), and RUNX1/2/3 (green). MYH11/RUNX overlap is shown in yellow. The number above each image refers to the unique patient identifier. All samples are from *CBFB*::*MYH11* patients. Note cytoplasmic MYH11 aggregates with RUNX colocalization.

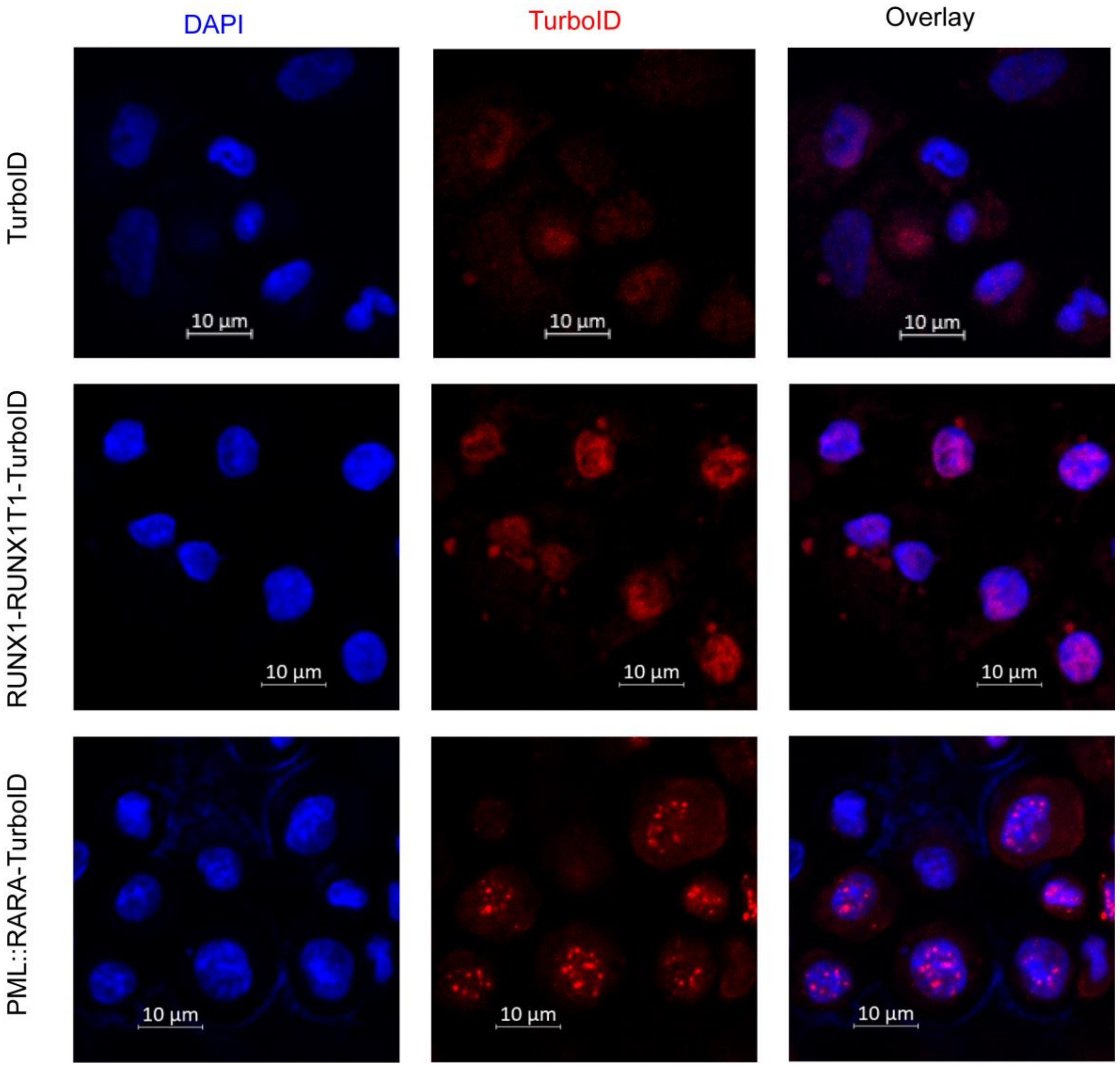

Figure S4. Immunofluorescence of primary murine hematopoietic cells transduced with the indicated TurboID fusion proteins. DAPI is shown in blue, and TurboID is shown in red. Note the localization of RUNX1::RUNX1T1-TurboID in the nucleus, and PML::RARA-TurboID in nuclear microspeckles.

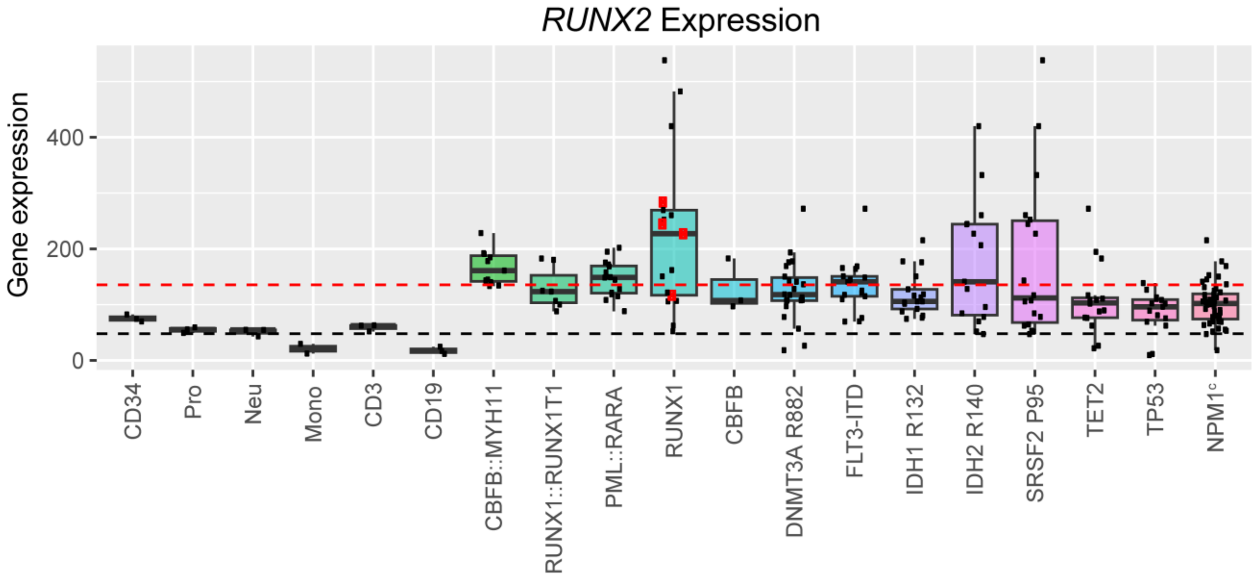

Figure S5. *RUNX2* gene expression levels in human AML from the TCGA AML study (1) with the indicated mutations/oncofusions. Black line indicates average *RUNX2* expression in healthy donor cells, and the dotted red line indicates the average *RUNX2* expression in AML cells. Red squares indicate biallelic *RUNX2* mutant samples.

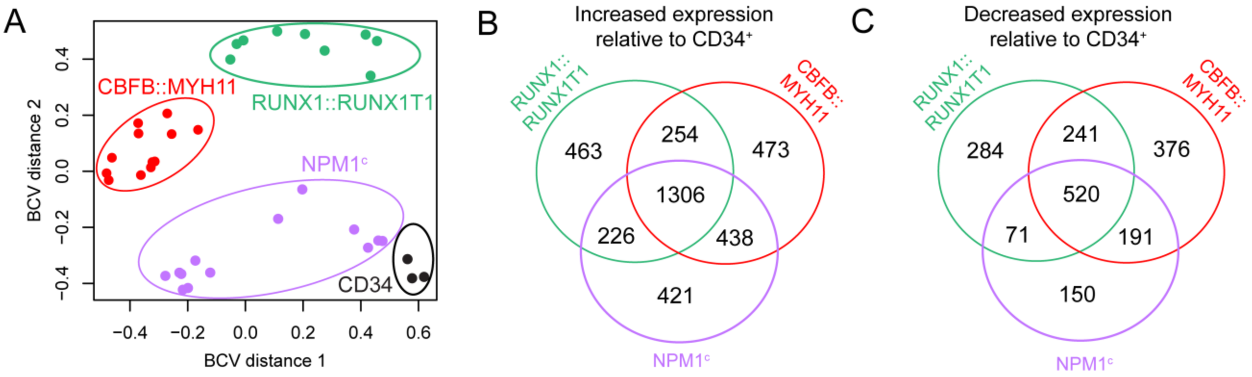

Figure S6. A) Multidimensional scaling plot of RNA-Seq data of validation cohort samples from **Figure 7E**). Note that each subgroup has a unique expression signature. B) and C) Venn diagrams of upregulated (B) and downregulated (C) genes relative to healthy donor CD34 cells. Note that *CBFB::MYH11* and *RUNX1::RUNX1T1* samples have distinct sets of dysregulated genes, but also, that most dysregulated genes are also shared with *NPM1*^{c} AMLs; many of these probably represent a more general AML-associated expression signature that is not specific for CBF AMLs.

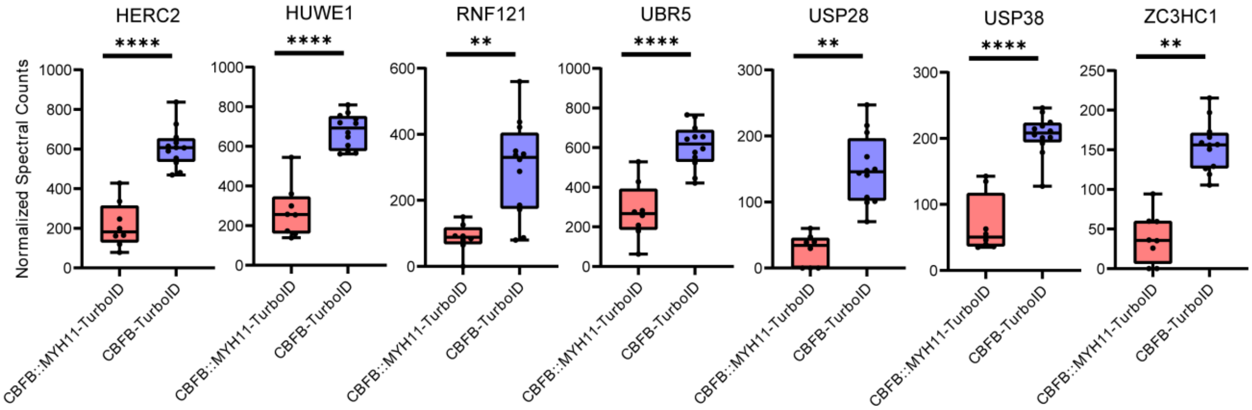

Figure S7. Normalized spectral counts of ubiquitin pathway-related differentially-interacting proteins between CBFB::MYH11-TurboID (red boxes, n=8) and CBFB-TurboID (blue boxes, n=12) in murine hematopoietic cells. Note increased interactions of several ubiquitin pathway proteins with CBFB relative to CBFB::MYH11. Unpaired t test, **p<0.01, ****p<0.0001.

Table S1: Normalized spectral counts for PML::RARA-TurboID differentially-interacting proteins versus TurboID alone

Table S2: Proteins in curated nuclear complexes. Protein names in red and bold indicate alternative gene names.

Table S3: Normalized spectral counts for RUNX1::RUNX1T1-TurboID differentially-interacting proteins versus TurboID alone

Table S4: Normalized spectral counts for CBFB::MYH11-TurboID differentially-interacting proteins versus TurboID alone

Table S5: Normalized spectral counts for CBFB-TurboID differentially-interacting proteins versus TurboID alone

Table S6: Normalized spectral counts for differentially interacting-proteins between CBFB-TurboID and CBFB::MYH11-TurboID

Table S7: Normalized RNA-Seq reads for differentially-expressed genes between murine hematopoietic cells transduced with *Empty Vector* MSCV-IRES-GFP (EV) or *CBFB*::*MYH11* MSCV-IRES-GFP (CM), with passive $CBFB^{N104A}$::*MYH11* MSCV-IRES-GFP samples.

Table S8: Normalized RNA-Seq reads for differentially-expressed genes between human healthy donor $CD34^+$ cells and human *RUNX1*::*RUNX1T1* AML samples, with passive *CBFB*::*MYH11* and $NPM1^c$-mutated AMLs.

Table S9: Normalized RNA-Seq reads for differentially-expressed genes between human healthy donor $CD34^+$ cells and human *CBFB*::*MYH11* AML samples, with passive *RUNX1*::*RUNX1T1* and $NPM1^c$-mutated AMLs.

Table S10: Normalized RNA-Seq reads for differentially-expressed genes between human healthy donor $CD34^+$ cells and human $NPM1^c$-mutated AML samples, with passive *RUNX1*::*RUNX1T1* and *CBFB*::*MYH11* AMLs.

Table S11: Human RNA-Seq samples

Table S12: Human AML immunofluorescence and Western blot samples

Repressor	Cells	Type	Species	Details	Ref
SIN3A	COS-7	Kidney fibroblast cell line	*Cercopithecus aethiops*	Co-transfected RUNX1 CBFB::MYH11 GAL4 fusion	(2, 3)
	ME-1	*CBFB*::*MYH11* leukemia cell line	*Homo sapiens*	Endogenous	(3, 4)
HDAC8	COS-7	Kidney fibroblast cell line	*Cercopithecus aethiops*	Co-transfected HDAC8 and CBFB::MYH11	(3)
	32Dcl2	Myeloid cell line	*Mus musculus*		(5)
	ME-1	*CBFB*::*MYH11* leukemia cell line	*Homo sapiens*	Interaction not detected	(3)
HDAC1	COS-7	Kidney fibroblast cell line	*Cercopithecus aethiops*	Co-transfected HDAC1 and CBFB::MYH11	(6)
	ME-1	*CBFB*::*MYH11* leukemia cell line	*Homo sapiens*	Co-transfected	(6)
	Primary AML	*Cbfb*::*Myh11* knock-in	*Mus musculus*	Endogenous	(6)
BMI-1	ME-1	*CBFB*::*MYH11* leukemia cell line	*Homo sapiens*	Endogenous	(4)
	HeLa	Cervical cancer cell line	*Homo sapiens*	Endogenous	(7)
RING1B	ME-1	CBFB::MYH11 leukemia cell line	*Homo sapiens*	Endogenous	(7)
	HeLa	Cervical cancer cell line	*Homo sapiens*	Endogenous	(7)

Table S13. Summary of reported interactions between CBFB::MYH11 and transcriptional repressors. None of these proteins were detected as CBFB::MYH11 interactors in primary mouse hematopoietic cells by TurboID proximity labeling (**Figure 2G**, **Table S4**).

Cells	Type	Species	Method	Localization	Notes	Ref
COS-7	Kidney fibroblast cell line	*Cercopithecus aethiops*	N/C split	Both	GAL4-tagged CBFB::MYH11	(2)
NIH3T3	Fibroblast cell line	*Mus musculus*	IF<br><br>N/C split	Nuclear<br><br>Cytoplasmic<br><br>Both	Requires RUNX1<br><br>Independent of RUNX1<br><br>Requires MYH11 C-terminus/ACD<br><br>Sequesters RUNX1	(2, 8–10)
Embryonic fibroblasts	Primary fibroblasts	*Mus musculus*	B-gal	Both	*Cbfb*::*Myh11* knock-in at *Cbfb* locus<br><br>Location inferred from RUNX1 location	(10)
Ba/F3	Pro-B cell line	*Mus musculus*	IF<br><br>N/C split	Nuclear<br><br>Both	RUNX1 required<br><br>MYH11 C-terminus/ACD required	(11–13)
32Dcl3	Myeloid cell line	*Mus musculus*	IF<br><br>N/C split	Nuclear		(5, 11)
Jurkat	T cell line	*Homo sapiens*	IF	Cytoplasmic		(14)
K562	Eyrthro-leukemia cell line	*Homo sapiens*	IF	Cytoplasmic		(15)
AML	Primary AML	*Homo sapiens*	IF<br><br>N/C split	Nuclear	Endogenous *CBFB*::*MYH11*	(16)
C2Cl2	Myoblast cell line	*Mus musculus*	IF	Cytoplasmic		(17)
293	Embryonic kidney	*Homo sapiens*	IF	Nuclear and cytoplasmic		(18)

Table S14. Summary of CBFB::MYH11 subcellular localization reported by immunofluorescence (IF) or nuclear-cytoplasmic fractionation and Western blotting (N/C split) organized by cell type. Note that nuclear-cytoplasmic fractionations have reported nuclear CBFB::MYH11 in cell lines with verified cytoplasmic CBFB::MYH11 by IF, attributed to precipitation of CBFB::MYH11 aggregates with nuclei (10).

Supplemental Video S1: 3D reconstruction of murine hematopoietic cell expressing CBFB::MYH11-GFP. Transduced cells were spun onto coverslips and allowed to recover normal shape overnight to minimize flattening artifacts. Cells were then stained for DAPI (blue), CBFB::MYH11-GFP (direct GFP imaging), and RUNX1 (red); CBFB::MYH11-GFP overlap with RUNX1 is shown in yellow. Serial confocal images were obtained, then reconstructed into a 3D video using Volocity software (Quorum Technologies).

Supplemental Video S2: 3D reconstruction of murine hematopoietic cell expressing CBFB^{N104A}::MYH11-GFP. Transduced cells were spun onto coverslips and allowed to recover normal shape overnight to minimize flattening artifacts. Cells were then stained for DAPI (blue), CBFB^{N104A}::MYH11-GFP (direct GFP imaging), and RUNX1 (red). Serial confocal images were obtained, then reconstructed into a 3D video using Volocity software (Quorum Technologies).

SUPPLEMENTARY METHODS

Proteomics Data Processing: Unprocessed data from the mass spectrometer were converted to peak lists using Proteome Discoverer (version 2.1.0.81, Thermo-Fischer Scientific). The MS2 spectra from peptides with +2, +3 and +4 charge states were analyzed using Mascot software(19) (Matrix Science, London, UK; version 2.5.1). Mascot was set up to search against a custom non-redundant database of mouse proteins (16,997 entries, based on mouse proteome download from Uniprot March 2021). The digestion enzyme was trypsin with a maximum of 4 missed cleavages allowed. The searches were performed with a fragment ion mass tolerance of 50 ppm and a parent ion tolerance of 25 ppm. Carbamidomethylation of cysteine was specified in Mascot as a fixed modification. Deamidation of asparagine, deamidation of glutamine, pyro-glutamate formation from n-terminal glutamine, acetylation of protein N-terminus and oxidation of methionine were specified as variable modifications. Peptides were filtered in Scaffold (version 5.2.1, Proteome Software Inc., Portland, OR) at 1% false-discovery rate (FDR) by searching against a reversed protein sequence database and a minimum of 2 peptides were required for protein identification.

WORKS CITED

1. Cancer Genome Atlas Research Network, et al. Genomic and epigenomic landscapes of adult de novo acute myeloid leukemia. *N Engl J Med*. 2013;368(22):2059–2074.

2. Lutterbach B, et al. The inv(16) encodes an acute myeloid leukemia 1 transcriptional corepressor. *Proc Natl Acad Sci U S A*. 1999;96(22):12822–12827.

3. Durst KL, et al. The inv(16) fusion protein associates with corepressors via a smooth muscle myosin heavy-chain domain. *Mol Cell Biol*. 2003;23(2):607–619.

4. Mandoli A, et al. CBFB-MYH11/RUNX1 together with a compendium of hematopoietic regulators, chromatin modifiers and basal transcription factors occupies self-renewal genes in inv(16) acute myeloid leukemia. *Leukemia*. 2014;28(4):770–778.

5. Qi J, et al. HDAC8 Inhibition Specifically Targets Inv(16) Acute Myeloid Leukemic Stem Cells by Restoring p53 Acetylation. *Cell Stem Cell*. 2015;17(5):597–610.

6. Richter LE, et al. HDAC1 Is a Required Cofactor of CBFβ-SMMHC and a Potential Therapeutic Target in Inversion 16 Acute Myeloid Leukemia. *Mol Cancer Res*. 2019;17(6):1241–1252.

7. Cordonnier G, et al. CBFβ-SMMHC Affects Genome-wide Polycomb Repressive Complex 1 Activity in Acute Myeloid Leukemia. *Cell Rep*. 2020;30(2):299-307.e3.

8. Wijmenga C, et al. Core binding factor beta-smooth muscle myosin heavy chain chimeric protein involved in acute myeloid leukemia forms unusual nuclear rod-like structures in transformed NIH 3T3 cells. *Proc Natl Acad Sci U S A*. 1996;93(4):1630–1635.

9. Lu J, et al. Subcellular localization of the alpha and beta subunits of the acute myeloid leukemia-linked transcription factor PEBP2/CBF. *Mol Cell Biol*. 1995;15(3):1651–1661.

10. Adya N, et al. The Leukemic Protein Core Binding Factor β (CBFβ)–Smooth-Muscle Myosin Heavy Chain Sequesters CBFα2 into Cytoskeletal Filaments and Aggregates. *Mol Cell Biol*. 1998;18(12):7432–7443.

11. Cao W, et al. CBF beta-SMMHC, expressed in M4Eo AML, reduced CBF DNA-binding and inhibited the G1 to S cell cycle transition at the restriction point in myeloid and lymphoid cells. *Oncogene*. 1997;15(11):1315–1327.

12. Cao W, et al. The core binding factor (CBF) alpha interaction domain and the smooth muscle myosin heavy chain (SMMHC) segment of CBFbeta-SMMHC are both required to slow cell proliferation. *J Biol Chem*. 1998;273(47):31534–31540.

13. Kummalue T, Lou J, Friedman AD. Multimerization via its myosin domain facilitates nuclear localization and inhibition of core binding factor (CBF) activities by the CBFbeta-smooth muscle myosin heavy chain myeloid leukemia oncoprotein. *Mol Cell Biol*. 2002;22(23):8278–8291.

14. Kanno Y, et al. Cytoplasmic Sequestration of the Polyomavirus Enhancer Binding Protein 2 (PEBP2)/Core Binding Factor α (CBFα) Subunit by the Leukemia-Related PEBP2/CBFβ-SMMHC Fusion Protein Inhibits PEBP2/CBF-Mediated Transactivation. *Mol Cell Biol*. 1998;18(7):4252–4261.

15. Liu P, et al. CBFB-MYH11 Fusion Sequesters RUNX1 in Cytoplasm to Prevent DNMT3A Recruitment to Target Genes in AML. *Front Cell Dev Biol*. 2021;9:1807.

16. Liu PP, et al. Identification of the chimeric protein product of the CBFB-MYH11 fusion gene in inv(16) leukemia cells. *Genes Chromosomes Cancer*. 1996;16(2):77–87.

17. Tanaka Y, et al. The chimeric protein, PEBP2 beta/CBF beta-SMMHC, disorganizes cytoplasmic stress fibers and inhibits transcriptional activation. *Oncogene*. 1998;17(6):699–708.

18. Zhao L, et al. The C-terminal multimerization domain is essential for leukemia development by CBFβ-SMMHC in a mouse knockin model. *Leukemia*. 2017;31(12):2841–2844.

19. Perkins DN, et al. Probability-based protein identification by searching sequence databases using mass spectrometry data. *Electrophoresis*. 1999;20(18):3551–3567.
